# Gd-metallofullerenol nanomaterial as non-toxic breast cancer stem cell-specific inhibitor

**DOI:** 10.1038/ncomms6988

**Published:** 2015-01-23

**Authors:** Ying Liu, Chunying Chen, Pengxu Qian, Xuefei Lu, Baoyun Sun, Xiao Zhang, Liming Wang, Xingfa Gao, Han Li, Zhiyun Chen, Jinglong Tang, Weijie Zhang, Jinquan Dong, Ru Bai, Peter E. Lobie, Qingfa Wu, Suling Liu, Huafeng Zhang, Feng Zhao, Max S. Wicha, Tao Zhu, Yuliang Zhao

**Affiliations:** 1 CAS Key Laboratory for Biomedical Effects of Nanomaterials and Nanosafety, National Center for Nanoscience and Technology of China and Institute of High Energy Physics, Chinese Academy of Sciences (CAS), Beijing 100190, China; 2 Hefei National Laboratory for Physical Sciences at Microscale and School of Life Sciences, University of Science and Technology of China, Hefei, Anhui 230027, China; 3 Cancer Science Institute of Singapore and Department of Pharmacology, National University of Singapore, Singapore 117456, Singapore; 4 Comprehensive Cancer Center, Department of Internal Medicine, University of Michigan, Ann Arbor, Michigan 48109, USA

## Abstract

The contemporary use of nanomedicines for cancer treatment has been largely limited to serving as carriers for existing therapeutic agents. Here, we provide definitive evidence that, the metallofullerenol nanomaterial Gd@C_82_(OH)_22_, while essentially not toxic to normal mammary epithelial cells, possesses intrinsic inhibitory activity against triple-negative breast cancer cells. Gd@C_82_(OH)_22_ blocks epithelial-to-mesenchymal transition with resultant efficient elimination of breast cancer stem cells (CSCs) resulting in abrogation of tumour initiation and metastasis. In normoxic conditions, Gd@C_82_(OH)_22_ mediates these effects by blocking TGF-β signalling. Moreover, under hypoxic conditions found in the tumour microenvironment, cellular uptake of Gd@C_82_(OH)_22_ is facilitated where it functions as a bi-potent inhibitor of HIF-1α and TGF-β activities, enhancing CSC elimination. These studies indicate that nanomaterials can be engineered to directly target CSCs. Thus, Gd-metallofullerenol is identified as a kind of non-toxic CSC specific inhibitors with significant therapeutic potential.

## Abstract (web summary)

A metallofullerenol nanomaterial, Gd@C_82_(OH)_22_, was shown to inhibit growth of several solid cancers in preclinical models and yet exhibit low toxicity. Herein the authors show that Gd@C_82_(OH)_22_ functions as an inhibitor of breast cancer stem cell function via blocking TGF-β and HIF-1α signalling, while sparing normal tissue.

## Graphical abstract

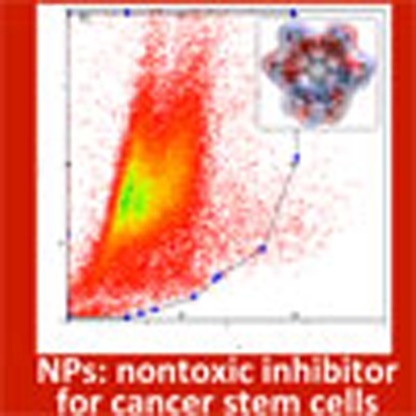

Compared with classic small-molecule drugs, nanomaterial-based nanomedicines are distinguished by their nanosizes and nanosurfaces that facilitate their interactions with biological systems at the nano/bio interface^1^. Nanomedicines hold great promise in medical applications especially in cancer therapeutics^2^. Currently, the predominant use of nanomaterials has been as carriers of conventional drugs, oligonucleotides or bioactive molecules where the nanomaterials may improve their bioavailability^3^. However, little evidence exists that nanomaterials themselves might possess intrinsic anticancer properties. We have previously reported the fullerene-based nanomaterial Gd@C_82_(OH)_22_, which is characterized by a rare earth atom gadolinium encapsulated by a cage consisting of 82 carbon atoms^4^,^5^. The surface of the carbon cage is modified with 22 hydroxyl groups to form Gd@C_82_(OH)_22_ with a virus-like morphological nanosurface^6^. With a size of ~1 nm, Gd@C_82_(OH)_22_ nanoparticles may aggregate by hydrogen bond interaction in a solution to form larger particles with sizes ranging from 20 to 120 nm, depending on the concentration and microenvironmental pH^1^. One of the most fascinating features of the Gd@C_82_(OH)_22_ nanoparticle is its strikingly low cyto- and systemic-toxicity despite a remarkable anticancer capacity in a variety of solid cancers^1^,^7^,^8^,^9^. However, the mechanisms by which Gd@C_82_(OH)_22_ nanoparticles mediate this cancer target specificity remain undefined.

Metastasis, chemotherapeutic resistance and recurrence are the major hurdles to successful treatment of cancer^10^,^11^. There is increasing evidence that these obstacles to clinically efficacious treatment may be mediated by a subpopulation of tumour cells that display stem cell properties. Although a number of approaches are being developed to target cancer stem cells (CSCs), as of yet, no single approach has proven efficacious^12^. Intra-tumoral heterogeneity as well as potential toxicity to normal tissues are important concerns that limit CSC-targeted therapeutics^10^,^12^,^13^. Herein, we utilized two claudin-low triple-negative breast cancer ((oestrogen receptor (ER), progesterone receptor (PR), no human epidermal growth factor receptor 2 (HER2) overexpression); TNBC) cell lines (MDA-MB-231 and BT549) that are enriched for features associated with epithelial-to-mesenchymal transition (EMT) and ‘breast cancer stem cell phenotypes’^14^,^15^,^16^. TNBC stands for a promiscuous group of breast cancer, and TNBC is also characterized by a high proportion of CSCs as assessed by expression of the CSC marker CD44^+^/CD24^−^ (ref. 17) or aldehyde dehydrogenase (ALDH)^18^.

Here we determined the mechanism by which Gd@C_82_(OH)_22_ nanoparticles effectively block EMT and reduce the CSC population in claudin-low breast cancer cell lines. Our studies provide the first definite evidence that a specific nanomaterial can selectively target CSC populations.

## Results

### Gd@C_82_(OH)_22_ treatment reverses the EMT phenotype

**Figure 1:**
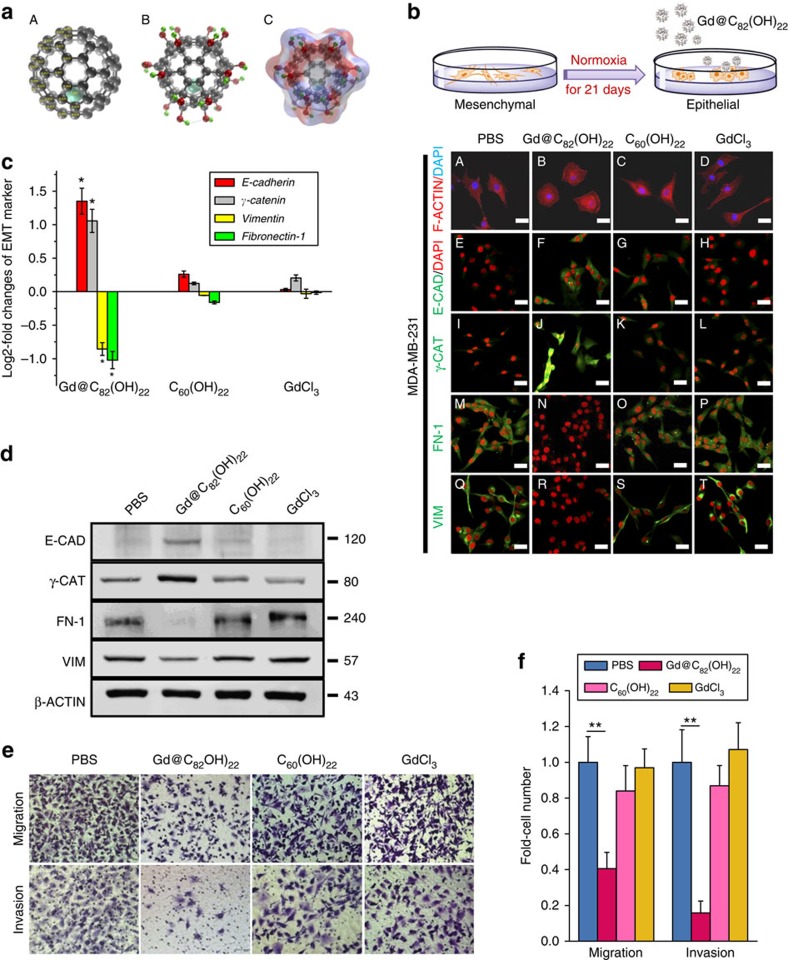
Treatment with Gd@C_82_(OH)_22_ impeded EMT, invasion and metastasis of TNBC cells *in vitro*. (**a**) Schematic drawing of Gd@C_82_(OH)_22_ nanoparticles. (A) Atomic structure of Gd@C_82_, which has a *C*_2v_ point group symmetry. Mülliken atomic charges of symmetrically unique atoms are marked. (B) Atomic structure of Gd@C_82_(OH)_22_. The dashed line indicates an intramolecular hydrogen bonds between hydrogen and carbon atoms. (C) Atomic structure of Gd@C_82_(OH)_22_ with colour on the molecular surface showing approximated electrostatic potential. (**b**) MDA-MB-231 cells were treated with PBS (A, E, I, M, Q), Gd@C_82_(OH)_22_ (B, F, J, N, R), C_60_(OH)_22_ (C, G, K, O, S) or GdCl_3_ (D, H, L, P, T) (all 50 μM) for 21 days. Phalloidin staining of F-ACTIN cytoskeleton (A–D) of cells in monolayer culture (scale bar, 12.5 μm) was visualized. (**c**) mRNA levels of EMT markers (*E-cadherin*, *γ-catenin*, *Vimentin* and *Fibronectin-1*) were analysed by qPCR (mean±s.e.m., *n*=3 each). *P<0.05 (ANOVA, Tukey’s *post-hoc* test). Protein levels of E-CADHERIN, γ-CATENIN, VIMENTIN and FIBRONECTIN-1 were detected by immunofluorescence staining (**b**) and western blot (**d**). Scale bar, 25 μm. (**e**,**f**) Cell migration and invasion were examined using trans-well cell culture chambers and Matrigel-coated ones (mean±s.e.m., *n*=6 each). **P<0.01 (one-way ANOVA, Tukey’s *post-hoc* test).

Gd@C_82_(OH)_22_ and C_60_(OH)_22_ nanoparticles synthesized as previously described have been well characterized^19^. As shown in Fig. 1a–A, Gd@C_82_(OH)_22_ possesses a lower *C*_2v_ symmetry compared with C_60_(OH)_22_. The carbon cage of Gd@C_82_(OH)_22_ receives electrons from the endohedral Gd atom, being in an anionic state. As a result of its lower symmetry, the C_82_ cage exhibits an inhomogeneous charge distribution. Accordingly, Gd@C_82_(OH)_22_ and C_60_(OH)_22_ were expected to react differently in the experimental hydroxylation processes, yielding fullerenols with distinct geometries and physical properties. Indeed, according to theoretical predictions the most thermodynamically stable structure of C_60_(OH)_22_ is that with all hydroxyl groups added aggregately to the equatorial region of the C_60_ cage^20^. In contrast, the theoretically predicted most stable structure of Gd@C_82_(OH)_22_ is that with hydroxyls around the C_82_ cage in a more homogenous and scattered manner (Fig. 1a–B,C)^21^. Such different geometries and the associated physical properties may serve as an origin of differential biological effects of Gd@C_82_(OH)_22_ and C_60_(OH)_22_.

Despite pronounced antitumour effects reported *in vivo*^1^,^7^,^8^,^9^, we observed that this nanoparticle exerted no significant effect on total TNBC cell proliferation or apoptosis *in vitro.* We treated triple-negative MDA-MB-231 human breast cancer cells with Gd@C_82_(OH)_22_, C_60_(OH)_22_, GdCl_3_ or PBS for extended periods. The ER-positive (ER+) MCF-7 cell line and immortalized but non-transformed MCF-10A human mammary epithelial cells were utilized as controls. Gd@C_82_(OH)_22_ and C_60_(OH)_22_ tended to aggregate in aqueous solutions (pH 7.0) and formed dispersed nanoparticles, respectively, with an average diameter of 100 nm^7^,^22^,^23^. No significant alteration in cell proliferation, as determined by the CCK-8 assay, was observed in any of the cell lines tested (days 3–21) (Supplementary Fig. 1a,c,e,g). Flow cytometric analysis with annexin V and PI double staining confirmed that Gd@C_82_(OH)_22_ exerted no appreciable effect on cell apoptosis/necrosis (Supplementary Fig. 1b,d,f,h).

Interestingly, Gd@C_82_(OH)_22_-treated MDA-MB-231 cells exhibited a less elongated morphology at day 14 compared with the PBS-treated cells. The Gd@C_82_(OH)_22_ promoted conversion of the spindle-like mesenchymal phenotype into a ‘cobble stone’ like epithelial phenotype was clearly observed after treatment for 21 days (Supplementary Fig. 2a). A limited conversion to an epithelial phenotype was observed in C_60_(OH)_22_-treated MDA-MB-231 cells, whereas the morphology of GdCl_3_- or PBS-treated cells remained unaltered (Fig. 1b,A–D). In comparison, no morphological alteration was observed in MCF-7 cells (Supplementary Fig. 3a) or MCF-10A cells (Supplementary Fig. 3c) treated with the same agents. Consistently, decreased branching colonies were only observed in MDA-MB-231 cells cultured on 2-dimensional, and in 3-dimensional matrigel with Gd@C_82_(OH)_22_ treatment (Supplementary Fig. 2a–B,F). Gd@C_82_(OH)_22_ modulation of cell morphology was concentration-dependent (Supplementary Fig. 4a). Colony scattering assays revealed a significantly larger proportion of compact cells and a smaller proportion of scattered cells on Gd@C_82_(OH)_22_ treatment (Supplementary Fig. 2a–J and Supplementary Fig. 2b). To determine the generality of this observation, we examined an additional TNBC cell line BT549. Consistently, an epithelial like conversion was only observed in BT549 cells treated with Gd@C_82_(OH)_22_ but not with other compounds or control (Supplementary Fig. 5a). Increased expression of epithelial makers (E-CADHERIN, γ-CATENIN) and reduced expression of mesenchymal markers (VIMENTIN, FIBRONECTIN-1) at both the mRNA (Fig. 1c) and protein levels (Fig. 1b,E–T,d) were observed in MDA-MB-231 cells treated with Gd@C_82_(OH)_22_ but not with the other compounds. Gd@C_82_(OH)_22_ treatment also resulted in increased expression of epithelial markers in mesenchymal like BT549 cells (Supplementary Fig. 5b) and epithelial like MCF-7 cells (Supplementary Fig. 3b) but not MCF-10A cells (Supplementary Fig. 3d). Functionally, MDA-MB-231 (Fig. 1e,f) and BT549 cells (Supplementary Fig. 5c,d) treated with Gd@C_82_(OH)_22_ displayed reduced wound closure (Supplementary Fig. 4b) as well as reduced migratory and invasive capacities.

Strikingly, after the removal of Gd@C_82_(OH)_22_ for 14 days (Supplementary Fig. 6), MDA-MB-231 cells maintained an epithelial morphology either in adherent culture or in 2D/3D culture for at least five more days (Supplementary Fig. 6a). Furthermore, MDA-MB-231 cells maintained reduced cell motility (Supplementary Fig. 6e,f) and a similar expression pattern of epithelial/mesenchymal markers (Supplementary Fig. 6d) following Gd@C_82_(OH)_22_ withdrawal. These studies demonstrate the sustained and potentially irreversible effects of Gd@C_82_(OH)_22_ treatment on TNBC cells.

### Gd@C_82_(OH)_22_ abrogates cell growth and metastasis

**Figure 2:**
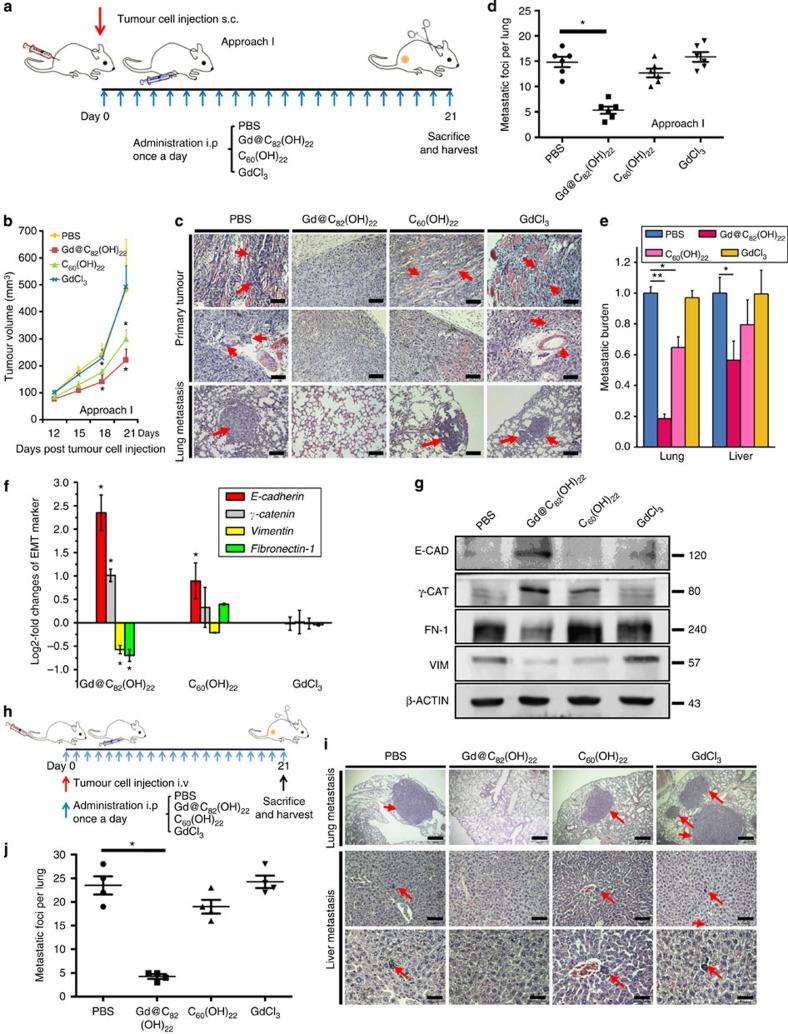
Gd@C_82_(OH)_22_ abrogates tumour growth and metastasis *in vivo*. (**a**) Schematic diagram of approach I (early treatment) in a mouse xenograft model. A total of 1 × 10^6^ MDA-MB-231 cells were injected s.c. and mice were treated either PBS or 2.5 μmol kg^−1^ Gd@C_82_(OH)_22_, C_60_(OH)_22_ or GdCl_3_ i.p., once a day, for 21 days. (**b**) Tumour growth curves in approach I mice were plotted (mean±s.e.m., *n*=5 each). *P<0.05 (two-way ANOVA, Bonferroni’s *post-hoc* test). (**c**) Sections from primary tumours and lungs were examined. Tumour cell invasion is shown in the top and middle panel and pulmonary micro-metastases are shown in the bottom panel. The arrows indicate metastatic tumour foci. (**d**) Pulmonary micro-metastases count (mean±s.e.m., *n*=10 each). *P<0.05 (one-way ANOVA, Tukey’s *post-hoc* test). (**e**) Relative expression of *hHPRT/mGAPDH* in the lung and liver tissues (mean±s.e.m., *n*=3 each). *P<0.05, **P<0.01 (one-way analysis of variance (ANOVA), Tukey’s *post-hoc* test). (**f**) mRNA levels of EMT markers in tumour tissues were analysed by qPCR (mean±s.e.m., *n*=3 each). *P<0.05 (one-way analysis of variance (ANOVA), Tukey’s *post-hoc* test). (**g**) Protein levels of E-CADHERIN (E-CAD), γ-CATENIN (γ-CAT), VIMENTIN (VIM) and FIBRONECTIN-1 (FN-1) in tumour tissues were detected by western blot. (**h**) Experimental design of the xenograft model with tumour cell tail vein injection (i.v.). A total of 1 × 10^6^ MDA-MB-231 cells were injected i.v., and mice were treated at day 0 for 21 days. (**i**) Pulmonary and hepatic micro-metastases of tumour cells. The arrows indicate metastatic tumour foci. (**j**) The lung micro-metastatic numbers were quantified (mean±s.e.m., *n*=6 each). *P<0.05 (one-way ANOVA, Tukey’s *post-hoc* test).

The effects of Gd@C_82_(OH)_22_ treatment on TNBC cell behaviour *in vivo* were investigated by two complimentary approaches. In the first approach (approach I, early treatment), 1 × 10^6^ MDA-MB-231 cells were injected *s.c.* into female BALB/c nude mice, followed by treatment (PBS, Gd@C_82_(OH)_22_ (2.5 μmol kg^−1^), C_60_(OH)_22_ (2.5 μmol kg^−1^) or GdCl_3_ (2.5 μmol kg^−1^)) once daily (Fig. 2a). Alternatively, in the second approach (approach II, terminal treatment), MDA-MB-231 cells were allowed to grow for 12 days to produce a tumour volume of ~100 mm^3^ and followed by once daily treatment (Supplementary Fig. 7a). Primary tumours (Supplementary Tables 1 and 2) and organ weights (the liver, spleen, kidney and lung) were measured on killing. Gd@C_82_(OH)_22_ significantly inhibited tumour growth by >50% in both approaches (Fig. 2b and Supplementary Fig. 7b).

In the first approach (early treatment), the accumulation of Gd in tumour tissues was 3.11±0.73 ng g^−1^ tumour wet weight. Pathological inspection indicated that primary tumours derived from MDA-MB-231 cells treated with PBS, C_60_(OH)_22_ or GdCl_3_ were poorly encapsulated and highly invasive with tumour emboli observed in lymphatic vessels. In contrast, tumours derived from the Gd@C_82_(OH)_22_-treated group remained well confined and noninvasive (Fig. 2c). Pulmonary and hepatic micro-metastases were quantified by qPCR analysis of the relative expression of *hHPRT*/*mGAPDH*. Both pulmonary and hepatic micrometastases were significantly reduced in the Gd@C_82_(OH)_22_-treated groups compared with the PBS-, C_60_(OH)_22_- or GdCl_3_-treated groups (Fig. 2d,e). Hence, Gd@C_82_(OH)_22_ inhibited both local invasion and distant metastasis of MDA-MB-231 cells. Consistently, tumours derived from the Gd@C_82_(OH)_22_-treated groups expressed higher levels of epithelial markers and lower levels of mesenchymal markers at both the mRNA (Fig. 2f) and protein levels (Fig. 2g and Supplementary Fig. 7c) compared with the other treatment groups. Tumour cell proliferation and apoptosis were also examined. The tumours derived from Gd@C_82_(OH)_22_-treated groups exhibited significantly less Ki-67-labelled cells (Supplementary Fig. 7d–f), but no significant changes were observed in the number of active-caspase-3-labelled cells (Supplementary Fig. 7g–i) compared with control tumours.

Pulmonary metastasis was also analysed in nude mice injected with 1 × 10^6^ MDA-MB-231 cells *via* the tail vein (Fig. 2h). The mice were treated with either PBS, Gd@C_82_(OH)_22_, C_60_(OH)_22_ or GdCl_3_ once daily. Metastatic tumours were readily detectable in the lungs of mice treated with either PBS (6/6), GdCl_3_ (6/6) or C_60_(OH)_22_ (5/6). In contrast, only one of six mice treated with Gd@C_82_(OH)_22_ developed pulmonary metastases (Supplementary Table 3). The incidence of micrometastatic deposits in either lung or liver tissue was also significantly lower in the Gd@C_82_(OH)_22_-treated groups compared with control treated groups (Fig. 2i,j).

### Gd@C_82_(OH)_22_ inhibits TGF-β to reduce EMT under normoxia

**Figure 3:**
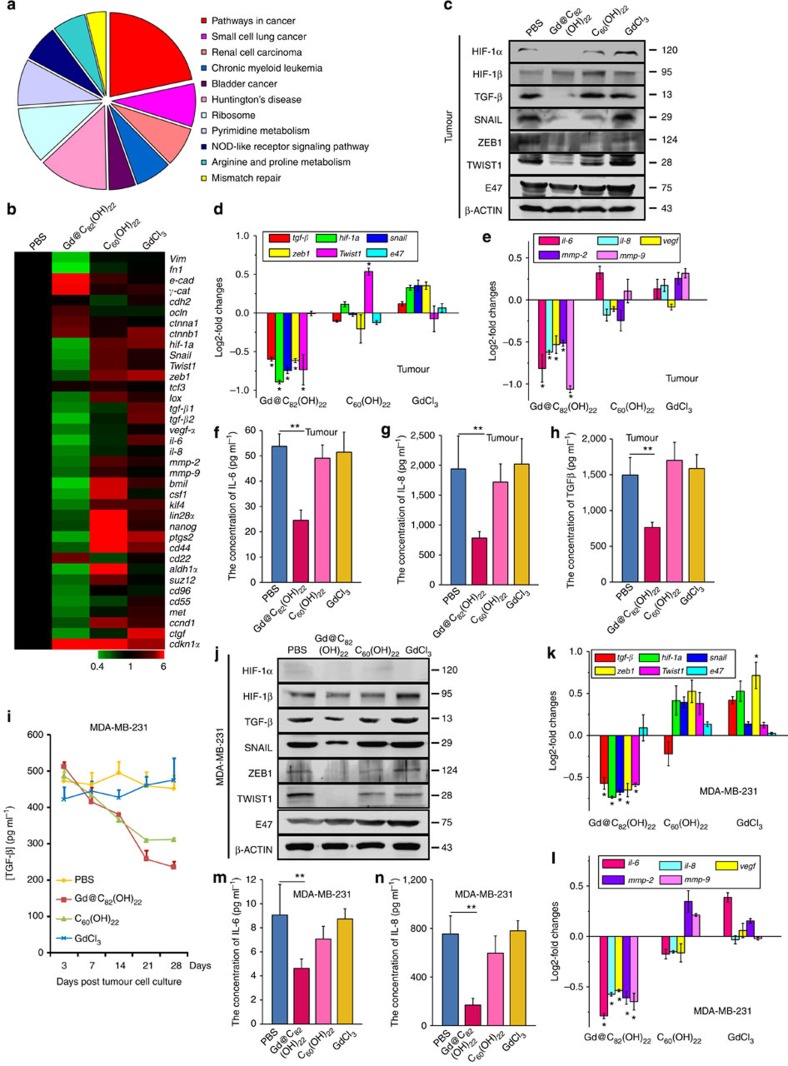
Pathway analysis of Gd@C_82_(OH)_22_ nanoparticles to impede EMT under normoxia. Cells were treated with PBS, Gd@C_82_(OH)_22_, C_60_(OH)_22_ or GdCl_3_ (all 50 μM) for 21 days. (**a**) KEGG pathway analysis of Gd@C_82_(OH)_22_ regulated genes by DAVID software. (**b**) Heat map depicting the mRNA expression profile of selected genes. Red squares correspond to increased expression, while green squares correspond to decreased expression of mRNA levels. MDA-MB-231 cells were subcutaneously injected into the mice at day 0. Mice were treated with either PBS or 2.5 μmol kg^−1^ Gd@C_82_(OH)_22_, C_60_(OH)_22_ or GdCl_3_ i.p., once a day before killing. (**c**) Protein levels of HIF-1α, HIF-1β, TGF-β, SNAIL, ZEB1, TWIST1 and E47 in tumour tissue were detected by western blot. (**d**) mRNA levels of *tgf*-β*, hif-1*α*, snail, zeb1, twist1* and *e47* in the tumour tissues were analysed by qPCR (mean±s.e.m., *n*=3 each). (**e**) mRNA levels of *il-6, il-8, mmp-2, mmp-9* and *vegf* in the tumour tissues were analysed by qPCR (mean±s.e.m., *n*=3 each). (**f**–**h**) ELISA analysis for expression of IL-6, (**f**) IL-8 (**g**) and TGF-β (**h**) in the serum was determined. All the data are represented as mean±s.e.m. (*n*=3 each) with *P<0.05 and **P<0.01 (one-way ANOVA, Tukey’s *post-hoc* test). MDA-MB-231 cells were treated with PBS, Gd@C_82_(OH)_22_, C_60_(OH)_22_ or GdCl_3_ for 21 days. (**i**) ELISA analysis for expression of TGF-β levels in the MDA-MB-231 culture medium treated with Gd@C_82_(OH)_22_ at day 3, 7, 14, 21 and 28 (mean±s.e.m., *n*=3 each). *P<0.05 (two-way ANOVA, Bonferroni’s *post-hoc* test). (**j**) Protein expression of HIF-1α, HIF-1β, TGF-β, SNAIL, ZEB1, TWIST1 and E47 were detected by western blot. (**k**) mRNA levels of *tgf*-β*, hif-1*α*, snail, zeb1, twist1* and *e47* were analysed by qPCR (mean±s.e.m., *n*=3 each). (**l**) mRNA levels of *il-6, il-8, mmp-2, mmp-9* and *vegf* were analysed by qPCR (mean±s.e.m., *n*=3 each). ELISA analysis for IL-6 (**m**) and IL-8 (**n**) concentrations in the supernatant derived from cell culture medium. All the data are represented as mean±s.e.m. (*n*=3 each) with *P<0.05 and **P<0.01 (one-way ANOVA, Tukey’s *post-hoc* test).

**Figure 4:**
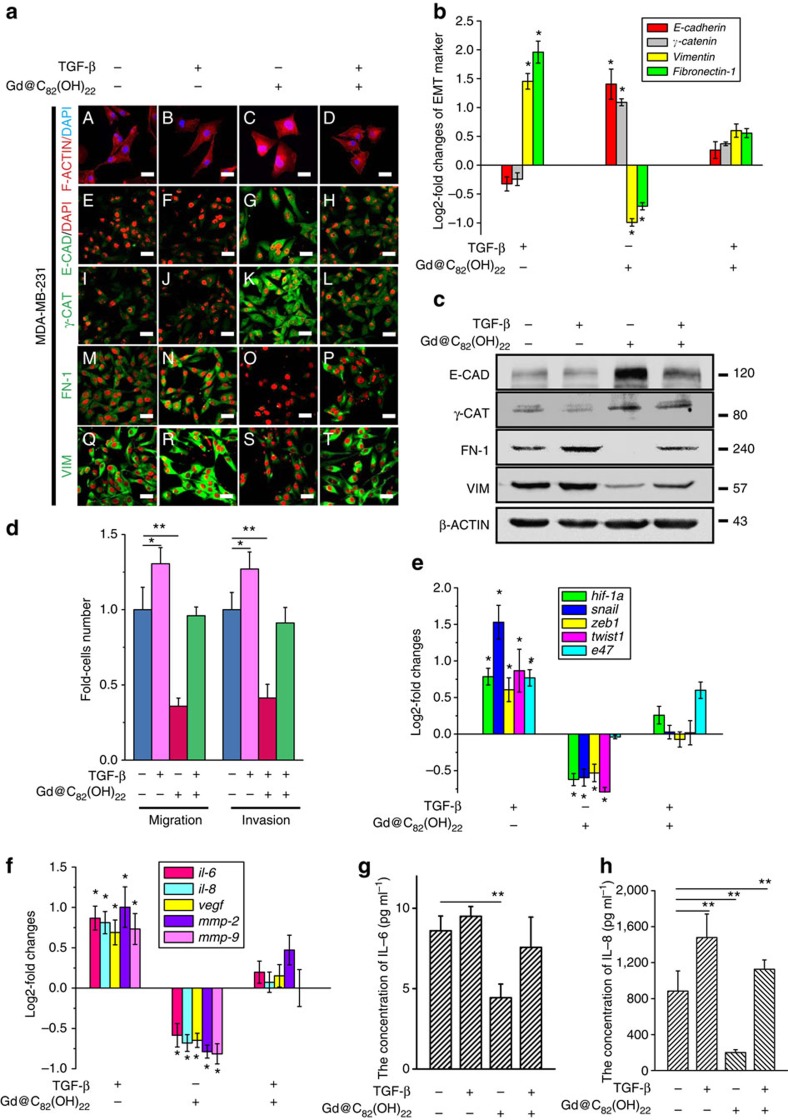
Gd@C_82_(OH)_22_ nanoparticles impeded EMT by the abrogation of TGF-β expression under normoxia. MDA-MB-231 cells were cultured with 20 ng ml^−1^ TGF-β supplement for 24 h after treatment with PBS or Gd@C_82_(OH)_22_ for 21 days. (**a**) Fluorescent visualization of F-ACTIN cytoskeleton was observed (A–D). Scale bar, 12.5 μm. E-T is the immunofluorescence staining of E-CADHERIN (E-CAD), γ-CATENIN (γ-CAT), VIMENTIN (VIM) and FIBRONECTIN-1 (FN-1). Scale bar, 25 μm. (**b**) mRNA levels *of E-cadherin*, *γ-catenin*, *Vimentin* and *Fibronectin-1* were analysed by qPCR (mean±s.e.m., *n*=3 each). *P<0.05 (two-way ANOVA, Bonferroni’s *post-hoc* test). (**c**) The protein expression of E-CADHERIN (E-CAD), γ-CATENIN (γ-CAT), VIMENTIN (VIM) and FIBRONECTIN-1 (FN-1) were detected by western blot. (**d**) Cell migration and invasion were examined using trans-well cell culture chambers and Matrigel-coated ones (mean±s.e.m., *n*=6 each). *P<0.05, **P<0.01 (two-way ANOVA, Bonferroni’s *post-hoc* test). (**e**) mRNA levels of *tgf*-β, *hif-1*α, *snail*, *zeb1*, *twist1* and *e47* were analysed by qPCR (mean±s.e.m., *n*=3 each). (**f**) mRNA levels of *il-6*, *il-8*, *mmp-2*, *mmp-9* and *vegf* were analysed by qPCR (mean±s.e.m., *n*=3 each). (**g**,**h**) ELISA analysis of IL-6 (**g**) and IL-8 (**h**) to determine their concentrations in the cell culture medium. All the data are represented as mean±s.e.m. (*n*=3 each) with *P<0.05 and **P<0.01 (two-way ANOVA, Bonferroni’s *post-hoc* test).

Recent evidence suggested that the tumour microenvironment regulates EMT that in turn generates CSCs. We therefore utilized Illumina next-generation sequencing and qPCR to examine the effects of the nanoparticles on the expression of EMT and CSC-related genes in MDA-MB-231 cells. Gd@C_82_(OH)_22_-responsive genes were mapped to KEGG pathways using the BLAST2GO software^24^, which identified tumour-associated pathways (Fig. 3a and Supplementary Fig. 8). A heat map depicting the mRNA expression profiles is shown in Fig. 3b. As indicated, Gd@C_82_(OH)_22_, but not the other treatments, significantly reduced the expression of genes associated with the mesenchymal phenotype as well as those associated with the CSCs. The expression levels of TGF-β and HIF-1α were significantly diminished by Gd@C_82_(OH)_22_, as were the EMT promoting zinc-finger proteins of the SNAIL families, ZEB1/2 and basic helix-loop-helix factor E47 (ref. 16). In addition, TGF-β, SNAIL, ZEB1, TWIST1 and pro-angiogenic factors (VEGF, IL-6, IL-8, MMP2, MMP9) were also downregulated at both the mRNA and protein levels in both Gd@C_82_(OH)_22_-treated cells and tumours derived from Gd@C_82_(OH)_22_-treated mice compared with control groups (Fig. 3c–h and Supplementary Fig. 7j). A decrease in secreted TGF-β in response to prolonged treatment of Gd@C_82_(OH)_22_ (Fig. 3i) was also observed. As expected, HIF-1α protein is undetectable in normoxia as oxygen exposure generates instability and rapid degradation^25^ (Fig. 3j). However, Gd@C_82_(OH)_22_ treatment still leads to a reduction of *hif-1*α mRNA (Supplementary Fig. 3k). Interestingly, Gd@C_82_(OH)_22_ also reduced the expression of CSC markers including *cd44* and *aldh1* as well as the CSC regulatory polycomb genes *bmi1* and *suz12* (Fig. 3b). We determined further whether the effects of Gd@C_82_(OH)_22_ on EMT were mediated by its modulation of TGF-β expression. As shown in Fig. 4a and Supplementary Fig. 2c,d, exogenous TGF-β abrogated the effects of Gd@C_82_(OH)_22_ on EMT. Reduced expression of E-CADHERIN and γ-CATENIN, and increased expression of VIMENTIN and FIBRONECTIN-1, at both the mRNA (Fig. 4b) and protein levels (Fig. 4a,c) was observed as a result. Consistently, the diminished cell motility due to Gd@C_82_(OH)_22_ treatment was rescued by exogenous TGF-β (Fig. 4d). Moreover, the mRNA levels of *tgf*-β, *hif-1*α, *snail*, *zeb1*, *twist1* and pro-angiogenic genes were also recovered to varying degrees as determined by qPCR (Fig. 4e–h). In summary, these results indicated that Gd@C_82_(OH)_22_ blocks EMT under normoxic conditions to a large extent via abrogation of TGF-β signalling.

### Gd@C_82_(OH)_22_ eliminates CSCs under normoxia

**Figure 5:**
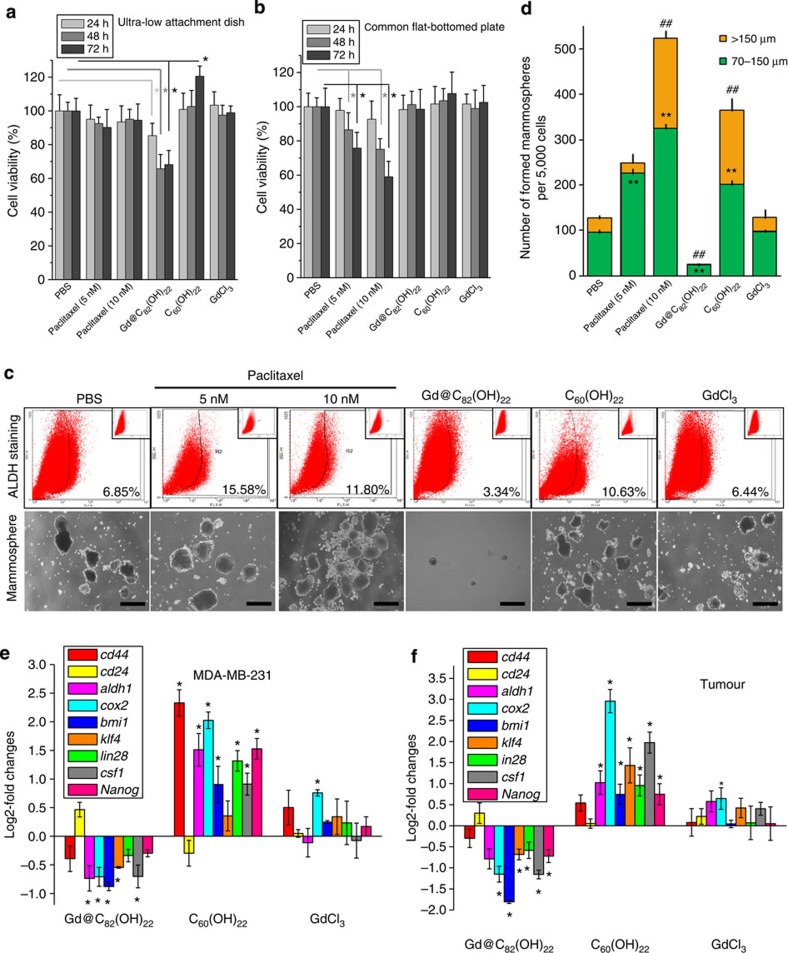
Gd@C_82_(OH)_22_ nanoparticles effectively eliminate CSCs and tumour initiation. (**a**,**b**) MDA-MB-231 cells were cultured in adherent monolayer culture or ultra-low attachment dish for 10 days, and treated with PBS, Paclitaxel (5 or 10 nM), Gd@C_82_(OH)_22_, C_60_(OH)_22_ or GdCl_3_ (all 50 μM) for another 24, 48 or 72 h. The cell viability was determined (mean±s.e.m., *n*=3 each). *P<0.05 (two-way ANOVA, Bonferroni’s *post-hoc* test). (**c**) Tumoursphere formation of MDA-MB-231 cells after treatment for 21 days (scale bar, 100 μm). (**d**) The tumourspheres were quantitated (mean±s.e.m., *n*=3 each). To tumourspheres of 70–150 μm, **P<0.01; to tumourspheres of >150 μm, ^##^*P*<0.01 (one-way ANOVA, Tukey’s *post-hoc* test). (**e**) mRNA levels of CSC markers were analysed by qPCR (mean±s.e.m., *n*=3 each). *P<0.05 (one-way ANOVA, Tukey’s *post-hoc* test). (**f**) A total of 1 × 10^6^ MDA-MB-231 cells were injected into the right back flanks of the mice s.c. Mice were monitored and treated daily. mRNA level of CSC markers in tumour tissue were analysed by qPCR (mean±s.e.m., *n*=3 each).

EMT programs have been inextricably associated with the acquisition of SC traits by normal and neoplastic cells^26^,^27^. Heat map data also shows that Gd@C_82_(OH)_22_ treatment decreased the expression of an array of stem cell makers (BMI1, CSF1, KLF4, LIN28A, NANOG) as well as CD44 and ALDH1 (Fig. 3b). We therefore determined whether Gd@C_82_(OH)_22_ could modulate CSC populations in TNBC. CSCs are enriched by growth as tumourspheres *in vitro*. MDA-MB-231 and BT549 cells were therefore cultured using ultra-low attachment dishes for 7–10 days to form tumourspheres. Dispersed cells were cultured on ultra-low attachment dishes for 3 days in the presence or absence of Gd@C_82_(OH)_22_ or the Paclitaxel. MDA-MB-231 and BT549 cells were also grown in adherent cultures in parallel. The proliferation of both MDA-MB-231 cells and BT549 cells in monolayer culture was markedly inhibited by Paclitaxel in a time- and dose-dependent manner as expected, whereas neither Gd@C_82_(OH)_22_ nor control agents affected cell growth (Fig. 5a and Supplementary Fig. 9a). However, the proliferation of stem cell-like cells derived from MDA-MB-231 and BT549 cells was rapidly inhibited by Gd@C_82_(OH)_22_ in suspension cultures. In comparison, treatment with Paclitaxel, PBS or GdCl_3_ did not affect tumoursphere formation under the utilized conditions (Fig. 5b and Supplementary Fig. 9b). Surprisingly, C_60_(OH)_22_ treatment promoted the proliferation of stem cell-like cells derived from MDA-MB-231 and BT549 cells.

**Figure 6:**
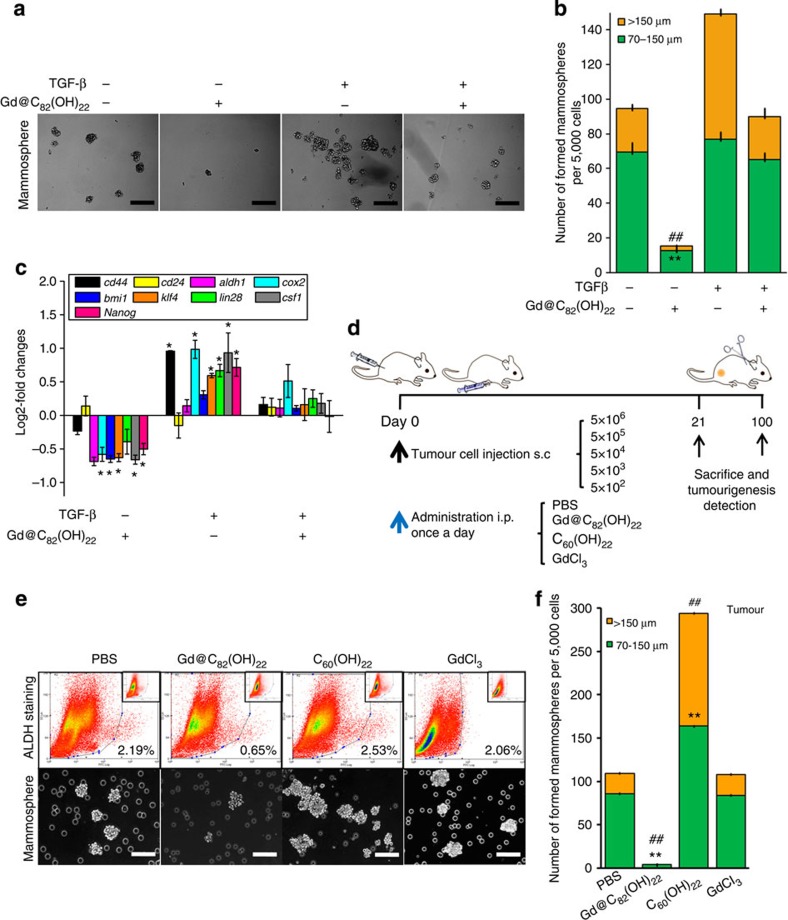
Gd@C_82_(OH)_22_ nanoparticles effectively eliminate CSCs by the abrogation of TGF-β under normoxia. (**a**) After treated with PBS, Gd@C_82_(OH)_22_, C_60_(OH)_22_ or GdCl_3_ for 21 days and 20 ng ml^−1^ TGF-β for 24 h, MDA-MB-231 tumourspheres were photographed and quantitated (**b**) (mean±s.e.m., *n*=3 each) (scale bar, 100 μm). To tumourspheres of 70–150 μm, **P<0.01; to tumourspheres of >150 μm, ^##^*P*<0.01 (two-way ANOVA, Bonferroni’s *post-hoc* test). (**c**) mRNA levels of CSC markers were analysed by qPCR (mean±s.e.m., *n*=3 each). *P<0.05 (two-way ANOVA, Bonferroni’s *post-hoc* test). (**d**) Tumour injection of MDA-MB-231 cells by limiting dilutions. Different numbers (5 × 10^6^, 5 × 10^5^, 5 × 10^4^, 5 × 10^3^, 5 × 10^2^) of MDA-MB-231 cells were injected into the right back flanks of the mice s.c. Mice were monitored and treated daily. (**e**) Tumoursphere formation of cells isolated from tumour tissues (mean±s.e.m., *n*=3 each). **P<0.01 (one-way ANOVA, Tukey’s *post-hoc* test). (**f**) The tumourspheres were counted.

We performed tumoursphere formation assays to further determine the influence of Gd@C_82_(OH)_22_ on the CSC population and properties in MDA-MB-231 and BT549 cells. Cells were treated with Gd@C_82_(OH)_22_ or controls for 21 days, or Paclitaxel for 7 days. Gd@C_82_(OH)_22_ potently diminished the number and size of tumourspheres formed in both TNBC cell lines compared with PBS treatment (Fig. 5c,d and Supplementary Fig. 9c,d). As expected, Paclitaxel promoted tumoursphere formation due to its selective toxicity to non-CSCs resulting in a relative enrichment of CSC and tumourspheres. Unexpectedly, C_60_(OH)_22_ significantly promoted tumoursphere formation and increased expression of SC markers compared with controls (Fig. 5e). Serial passaging experiments are the accepted methodology for determining the self-renewal capacity of CSCs^28^,^29^. We observed that Gd@C_82_(OH)_22_ significantly inhibited primary and secondary tumoursphere formation of MDA-MB-231 cells (Supplementary Fig. 10a,b). In contrast, Gd@C_82_(OH)_22_ did not affect mammosphere formation of normal MCF-10A cells (Supplementary Fig. 10c,d). We further determined the role of TGF-β in mediating the effects of Gd@C_82_(OH)_22_ on the CSC populations. After treatment with PBS, Gd@C_82_(OH)_22_, C_60_(OH)_22_ or GdCl_3_ for 21 days, MDA-MB-231 cells were cultured with 20 ng ml^−1^ TGF-β for 24 h. Gd@C_82_(OH)_22_ repression of tumoursphere formation, and cells expressing CSC markers, was significantly rescued by TGF-β treatment (Fig. 6a–c).

CSCs can be isolated from mammary carcinomas by virtue of their increased expression of ALDH as assessed by the ALDEFLUOR assay^30^. Although CD44+/CD24− and ALDH+ as the most often used molecular markers for breast CSCs, not all breast cancer cell lines contain CSCs equivalently expressing these markers. The proportion of CD44+/CD24− cells in MDA-MB-231 was about 80% (PBS 84.73%, Gd@C_82_(OH)_22_ 80.6%, C_60_(OH)_22_ 86.6%, GdCl_3_ 85.77%), which rendered these markers unsuitable for use in this cell line. As a result, ALDH was utilized.

Treatment with Gd@C_82_(OH)_22_ in MDA-MB-231 and BT549 cells reduced the percentage of ALDH+ cells by more than twofold, whereas C_60_(OH)_22_ treatment increased the percentage of ALDH+ cells by ~50% (Fig. 5c). As expected, Paclitaxel treatment led to a substantial increase in the percentage of ALDH+ cells. Consistently decreased expression of SC markers (*cd44*, *aldh1*, *cox2*, *bmi1*, *klf4*, *lin28*, *csf1* and *nanog*) and increased expression of *cd24*, as a result of Gd@C_82_(OH)_22_ treatment of MDA-MB-231, was observed (Fig. 5e). In contrast, C_60_(OH)_22_ increased expression of SC markers and decreased expression of CD24 as determined by qPCR analysis. Together, these results demonstrated that Gd@C_82_(OH)_22_ effectively repressed breast CSC properties *in vitro*, whereas C_60_(OH)_22_ promoted breast CSC traits.

To ascertain the effect of Gd@C_82_(OH)_22_ on tumour-initiating capacity *in vivo*, we injected MDA-MB-231 cells subcutaneously into nude mice at a series of limiting dilutions from 5 × 10^6^ to 500 cells and treated with Gd@C_82_(OH)_22_ or controls and determined tumour formation. As summarized in Fig. 6d, in a period of 100 days after tumour cell injection, the group of mice injected with 5,000 or 500 cells did not form any tumours with Gd@C_82_(OH)_22_ treatment. In contrast, inoculation of the equivalent numbers of cells in PBS-, C_60_(OH)_22_- and GdCl_3_-treated groups resulted in significant tumour formation (Supplementary Table 4) (Table 1). The tumours were collected to isolate primary tumour cells for the ALDEFLUOR assay and tumoursphere formation. The proportion of ALDH+ cells in the Gd@C_82_(OH)_22_-treated group was significantly less than PBS-treated group (0.65% versus 2.19%) (Fig. 6e), further verifying the capacity of Gd@C_82_(OH)_22_ to diminish the CSC population *in vivo.* Furthermore, the number and size of tumourspheres generated from (Fig. 6e,f), and the expression levels of SC markers, cells derived from Gd@C_82_(OH)_22_-treated tumours were significantly reduced compared with PBS controls (Fig. 5f). Collectively, these observations indicate that Gd@C_82_(OH)_22_ treatment significantly reduces the tumour-initiating CSC population in TNBC.

### Gd@C_82_(OH)_22_ inhibits HIF-1α and TGF-β under hypoxia

**Figure 7:**
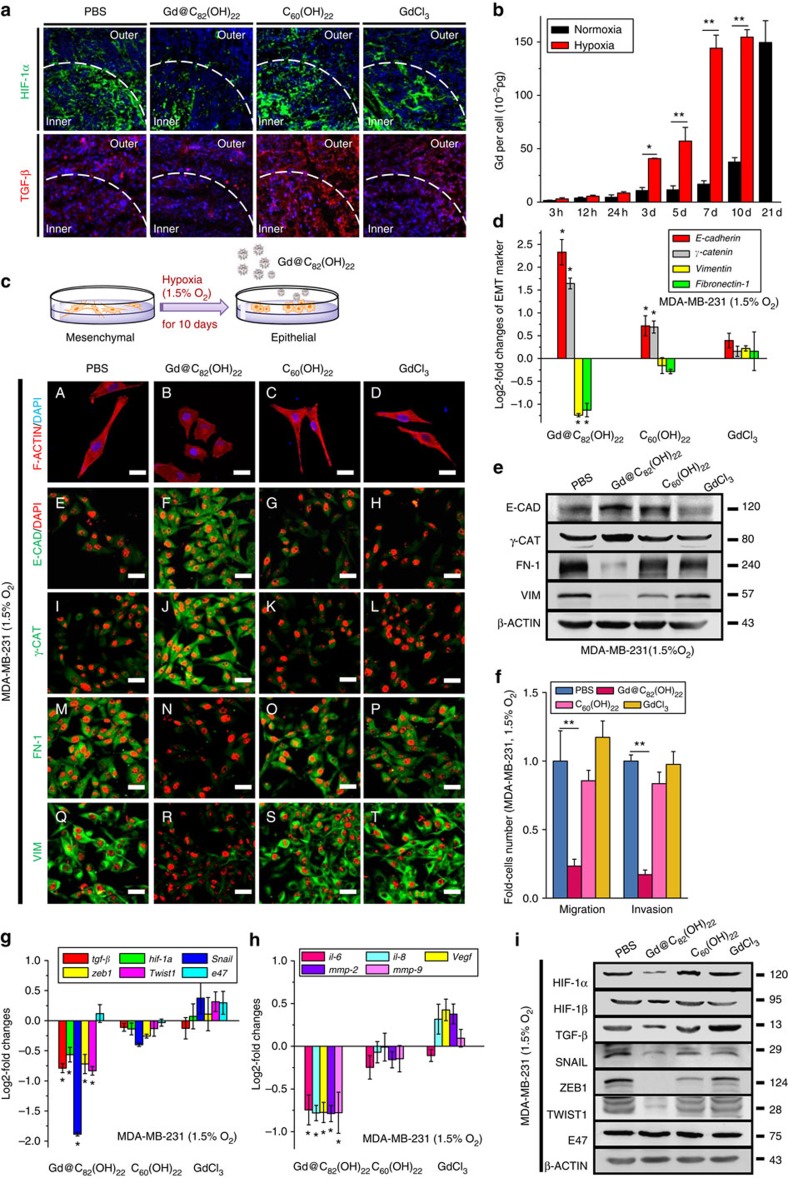
Gd@C_82_(OH)_22_ impaired EMT under hypoxia. A total of 1 × 10^6^ MDA-MB-231 cells were injected s.c. and mice were treated at day 0 for 21 days. (**a**) HIF-1α and TGF-β expression in the periphery and inner portions of tumours. Scale bar, 20 μm. (**b**) The amount of intracellular Gd after MDA-MB-231 cells were treated with 50 μM Gd@C_82_(OH)_22_ under hypoxia and normoxia were detected by ICP-MS (mean±s.e.m., *n*=3 each). *P<0.05, **P<0.01 (two-way ANOVA, Bonferroni’s *post-hoc* test). (**c**) MDA-MB-231 cells were cultured under hypoxia and treated with PBS (A, E, I, M, Q), 50 μM Gd@C_82_(OH)_22_ (B, F, J, N, R), C_60_(OH)_22_ (C, G, K, O, S) and GdCl_3_ (D, H, L, P, T) for 10 days. (A–D) F-ACTIN cytoskeleton of cells (scale bar, 12.5 μm). Immunofluorescence staining (**c**, E-T), qPCR (**d**) and western blot analysis (**e**) of E-CADHERIN (E-CAD), γ-CATENIN (γ-CAT), VIMENTIN (VIM) and FIBRONECTIN-1 (FN-1) of MDA-MB-231 cells under hypoxia. (**f**) Cell migration and invasion were examined using trans-well cell culture chambers and Matrigel-coated ones (mean±s.e.m., *n*=6 each). **P<0.01 (one-way ANOVA, Tukey’s *post-hoc* test). mRNA levels of *hif-1*α, *hif-1*β, *tgf*-β, *snail*, *zeb1*, *twist1*, *e47* (**g**), *il-6*, *il-8*, *mmp-2*, *mmp-9* and *vegf* (**h**) were analysed by qPCR (mean±s.e.m., *n*=3 each). (**i**) Protein levels of HIF-1α, HIF-1β, TGF-β, SNAIL, ZEB1, TWIST1 and E47 were detected by western blot.

In solid tumours, decreased oxygen and nutrient supply creates a hypoxic microenvironment in the central region of tumours. Hypoxia has previously been shown to increase the CSC population in a process mediated by HIF-1α, a major response gene to tissue hypoxia^25^,^31^. HIF-1α was readily detectable in tumour sections by immunofluoresence. We further demonstrated that Gd@C_82_(OH)_22_ effectively inhibited the expression of HIF-1α and TGF-β in tumours derived from MDA-MB-231 cells treated with Gd@C_82_(OH)_22_ compared with controls in mouse xenografts (Fig. 7a). Interestingly, HIF-1α expression was significantly increased in the inner portions of tumour sections compared with the tumour periphery. In contrast, there was no detectable difference in the intensity of TGF-β staining between the periphery and inner portions of consecutive sections in the control tumours or those treated with the nanoparticles. As accumulating evidence suggests that intratumoral hypoxia and TGF-β promote tumour metastasis^32^, we determined whether Gd@C_82_(OH)_22_ repression of HIF-1α or TGF-β signalling is responsible for inhibition of EMT by this compound.

**Figure 8:**
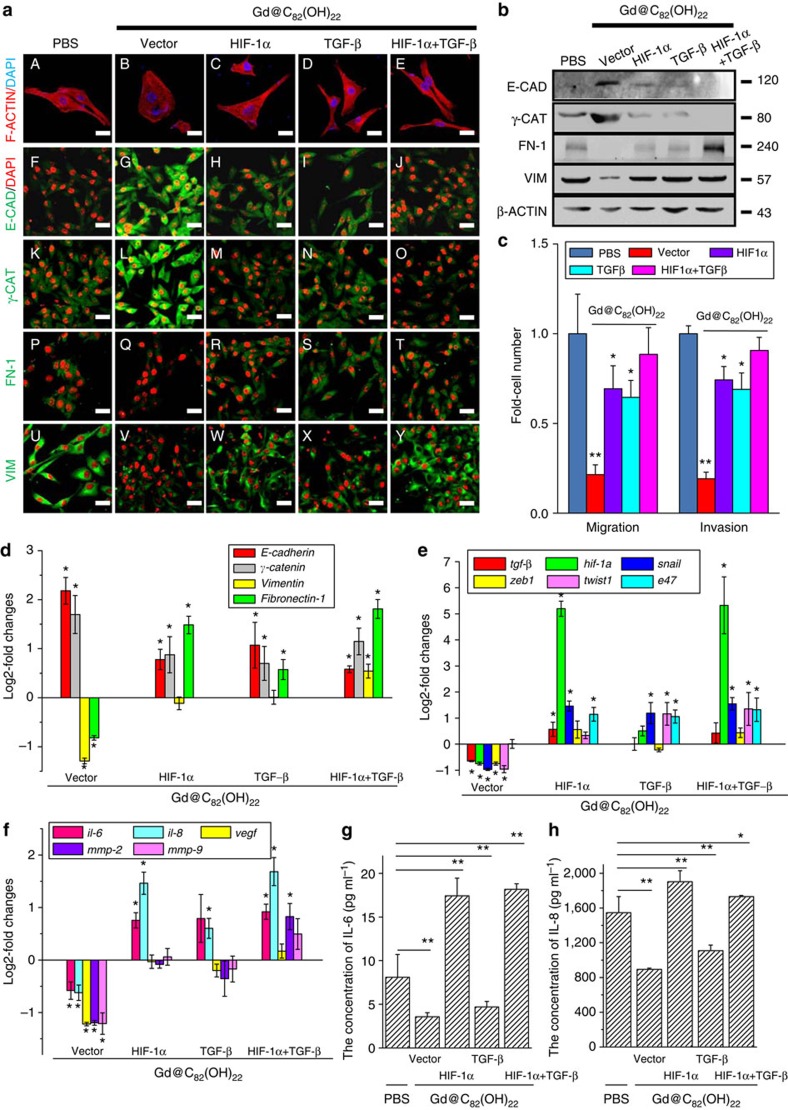
Gd@C_82_(OH)_22_ impaired EMT by inhibition of TGF-β and HIF-1α expression under hypoxia. MDA-MB-231 cells were transfected with *hif-1*α expressing plasmid and/or treated with 20 ng ml^−1^ TGF-β with further culture in the presence of Gd@C_82_(OH)_22_ or PBS under hypoxia for 10 days. (**a**; A-E) F-ACTIN cytoskeleton of cells (scale bar, 12.5 μm). Immunofluorescence staining (**a**, F-Y) and western blot analysis (**b**) of E-CADHERIN (E-CAD), γ-CATENIN (γ-CAT), VIMENTIN (VIM) and FIBRONECTIN-1 (FN-1). Scale bar, 25 μm. (**c**) The migratory and invasive cells were examined using trans-well cell culture chambers and Matrigel-coated ones (mean±s.e.m., *n*=3 each). *P<0.05 and **P<0.01 (two-way ANOVA, Bonferroni’s *post-hoc* test). mRNA levels of EMT markers (**d**), *hif-1*α, *hif-1*β, *tgf*-β, *snail*, *zeb1*, *twist1*, *e47* (**e**), *il-6*, *il-8*, *mmp-2*, *mmp-9* and *vegf* (**f**) were analysed by qPCR (mean±s.e.m., *n*=3 each). ELISA analysis for expression of IL-6 (**g**) and IL-8 (**h**) in the MDA-MB-231 cell culture medium was determined. All the data are represented as mean±s.e.m. (*n*=3 each) with *P<0.05 and **P<0.01 (two-way ANOVA, Bonferroni’s *post-hoc* test).

Using inductively coupled plasma mass spectrometry (ICP-MS), we observed that Gd@C_82_(OH)_22_ nanoparticles can be rapidly internalized by MDA-MB-231 cells under hypoxic conditions. In comparison, the cellular uptake of Gd@C_82_(OH)_22_ nanoparticles under normoxia was significantly lower compared with that observed under hypoxia in the first 10 days of treatment. The average Gd concentration in cells cultured under hypoxia for 10 days was similar to that under normoxia for 21 days (Fig. 7b). In hypoxic conditions, Gd@C_82_(OH)_22_-treated cells exhibited a compact, cobblestone-like epithelial phenotype, whereas PBS-, C_60_(OH)_22_- or GdCl_3_-treated cells retained a mesenchymal spindle-like morphology (Fig. 7c, Supplementary Fig. 2e,f and Supplementary Fig. 11a). These results demonstrated that hypoxia facilitates Gd@C_82_(OH)_22_ uptake resulting in repression of the EMT phenotype. The altered expression of epithelial and mesenchymal markers at the mRNA (Fig. 7d) and protein levels (Fig. 7c,e, IHC & WB) in Gd@C_82_(OH)_22_-treated cells compared with control cells under hypoxia further support the phenotypic changes observed. Functionally, cell migration and invasion were markedly inhibited by Gd@C_82_(OH)_22_ compared with controls (Fig. 7f, Supplementary Fig. 11b,c). We further demonstrated that under hypoxic conditions Gd@C_82_(OH)_22_ significantly reduced the expression of TGF-β, HIF-1α, SNAIL, ZEB1, TWIST1 and pro-angiogenic factors (VEGF, IL-6, IL-8, MMP2, MMP9) at the mRNA (Fig. 7g,h) and protein levels (Fig. 7i and Supplementary Fig. 11d–f) when compared with controls. In contrast, Gd@C_82_(OH)_22_ treatment did not affect total cell proliferation or apoptosis or necrosis under hypoxia indicating a lack of cytotoxicity towards the bulk cell population (Supplementary Fig. 12). To further determine whether the effects of Gd@C_82_(OH)_22_ are mediated by repression of HIF-1α and TGF-β, we transiently transfected MDA-MB-231 cells with a HIF-1α expressing plasmid in the presence or absence of exogenous TGF-β. Cells were maintained under hypoxia with 50 μM Gd@C_82_(OH)_22_ or PBS treatment for 10 days. As shown in Fig. 8a–c and Supplementary Fig. 2g,h, forced expression of HIF-1α or TGF-β supplementation partially reversed Gd@C_82_(OH)_22_ repression of cell migration and invasion. The expression levels of TGF-β, HIF-1α and a series of EMT markers, which were diminished by Gd@C_82_(OH)_22_ treatment, were all significantly recovered by HIF-1α overexpression and/or TGF-β treatment (Fig. 8d–h). These results suggest that Gd@C_82_(OH)_22_ nanoparticles block EMT in hypoxia through abrogation of TGF-β and HIF-1α expression.

### Gd@C_82_(OH)_22_ eliminates CSCs under hypoxia

**Figure 9:**
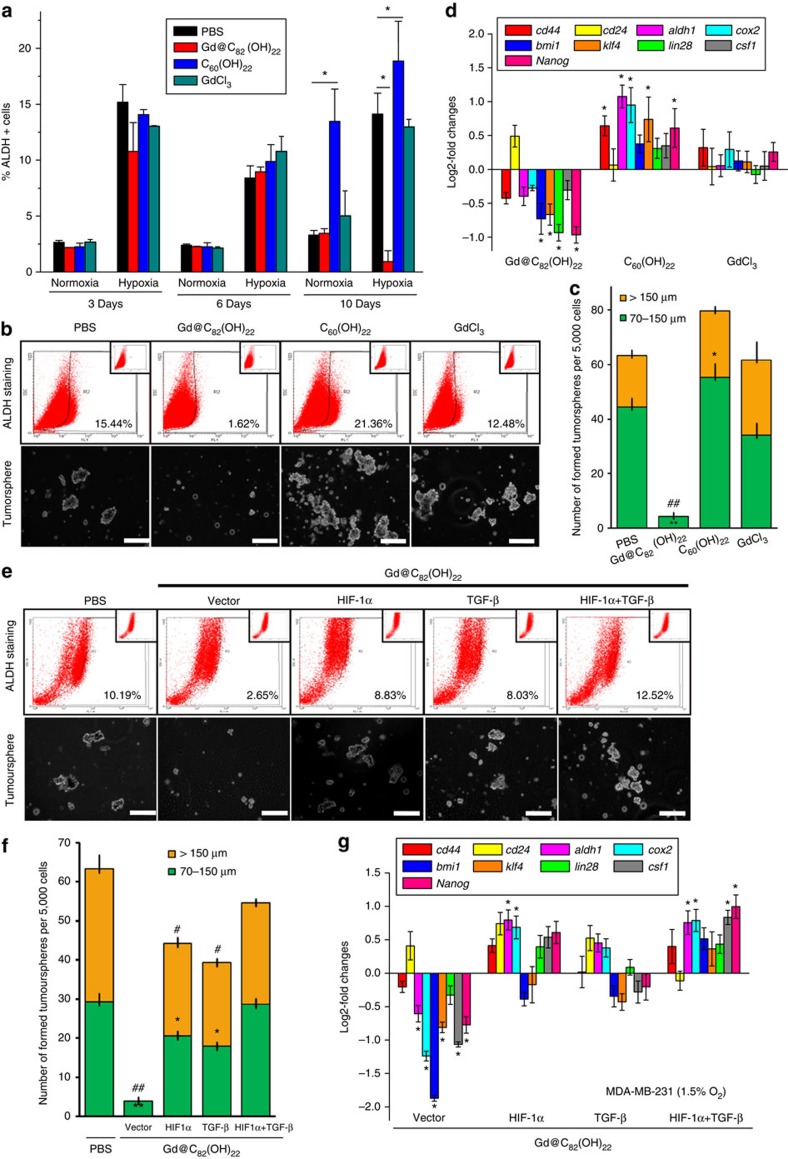
Gd@C_82_(OH)_22_ efficiently eliminated breast CSC population through TGF-β and HIF-1a. MDA-MB-231 cells were maintained in PBS, Gd@C_82_(OH)_22_, C_60_(OH)_22_ or GdCl_3_ under hypoxia for 10 days for ALDEFLUOR (**a**) and tumoursphere formation assay (**b**) (scale bar, 100 μm) (mean±s.e.m., *n*=3 each). *P<0.05 (two-way ANOVA, Bonferroni’s *post-hoc* test). (**c**) The tumourspheres count (mean±s.e.m., *n*=3 each). To tumourspheres of 70–150 μm, *P<0.05 and **P<0.01; to tumourspheres of > 150 μm, ^#^*P*<0.05 and ^##^*P*<0.01 (one-way ANOVA, Tukey’s *post-hoc* test). (**d**) The mRNA levels of CSC markers were analysed by qPCR (mean±s.e.m., *n*=3 each). **P<0.01 (one-way ANOVA, Tukey’s *post-hoc* test). (**e**) Tumoursphere formation assay after MDA-MB-231 cells were transfected with HIF-1α expressing plasmid and/or supplemented with 20 ng ml^−1^ TGF-β and cultured under hypoxia for 10 days (scale bar, 12.5 μm). (**f**) The tumourspheres count (mean±s.e.m., *n*=3 each). To tumourspheres of 70–150 μm, *P<0.05 and **P<0.01; to tumourspheres of >150 μm, ^#^*P*<0.05 and ^##^*P*<0.01 (two-way ANOVA, Bonferroni’s *post-hoc* test). (**g**) The mRNA levels of CSC markers were analysed by qPCR (mean±s.e.m., *n*=3 each). *P<0.05 (two-way ANOVA, Bonferroni’s *post-hoc* test).

We next utilized the ALDEFLUOR and tumoursphere assays to determine the efficacy of Gd@C_82_(OH)_22_ on CSC populations of MDA-MB-231 cells grown under hypoxic conditions. MDA-MB-231 cells were treated with PBS, Gd@C_82_(OH)_22_, C_60_(OH)_22_ or GdCl_3_ under hypoxia or normoxia for 3, 6 and 10 days. Consistent with recent reports that intratumoral hypoxia increases the CSC populations^31^,^33^, MDA-MB-231 cells under hypoxia exhibited a significantly increased ALDH expressing cell population (Fig. 9a) compared with cells cultured in normoxia at all time points. Gd@C_82_(OH)_22_ treatment of MDA-MB-231 under hypoxia for 10 days markedly reduced the percentage of ALDH+ cells by more than 11-fold, whereas Gd@C_82_(OH)_22_ treatment under normoxia up to 10 days did not alter the percentage of ALDH+ cells. In contrast, C_60_(OH)_22_ treatment under both hypoxia and normoxia for 10 days significantly increased the percentage of ALDH+ cells (Fig. 9a). To demonstrate that ALDH is a reliable marker for CSCs under hypoxia, we counted total cell numbers when the cells were collected for ALDEFLUOR assay. A total of 1 × 10^6^ cells from either control group or Gd@C_82_(OH)_22_ group were utilized for the ALDEFLUOR assay. On the basis of the total cell number and the percentage of ALDH+ cells from each group, the absolute number of ALDH+ cells was quantitated (Control group: (14.74±0.94) × 10^4^, Gd@C_82_(OH)_22_ group: (1.22±0.43) × 10^4^), indicating decreased CSC numbers rendered by Gd@C_82_(OH)_22_ under hypoxia. Consistently, Gd@C_82_(OH)_22_ treatment under hypoxia for 10 days markedly inhibited tumoursphere formation and mRNA expression of CSC markers (Fig. 9d), whereas C_60_(OH)_22_ treatment increased tumoursphere formation (Fig. 9b,c).

As HIF-1α and TGF-β have been identified as two critical targets of Gd@C_82_(OH)_22_, we further determined whether these molecules also mediate Gd@C_82_(OH)_22_ exerted effects on CSCs under hypoxia. MDA-MB-231 cells were maintained in 50 μM Gd@C_82_(OH)_22_ under hypoxia for 10 days, and simultaneously transfected with a HIF-1α-expressing plasmid or control vector, and/or cultured with 20 ng ml^−1^ TGF-β. The size and number of tumourspheres, the percentage of ALDH+ cells and the expression of CSC markers, which had been repressed by Gd@C_82_(OH)_22_, were rescued by forced expression of HIF-1α and/or TGF-β treatment (Fig. 9e–g). Of note, a combination of HIF-1α overexpression and TGF-β was required for maximum rescue of the cellular alterations produced by Gd@C_82_(OH)_22_ treatment. In aggregate, these data suggest that Gd@C_82_(OH)_22_ nanoparticles eliminated breast cancer cells via simultaneous repression of TGF-β and HIF-1α signalling under hypoxia.

## Discussion

**Figure 10:**
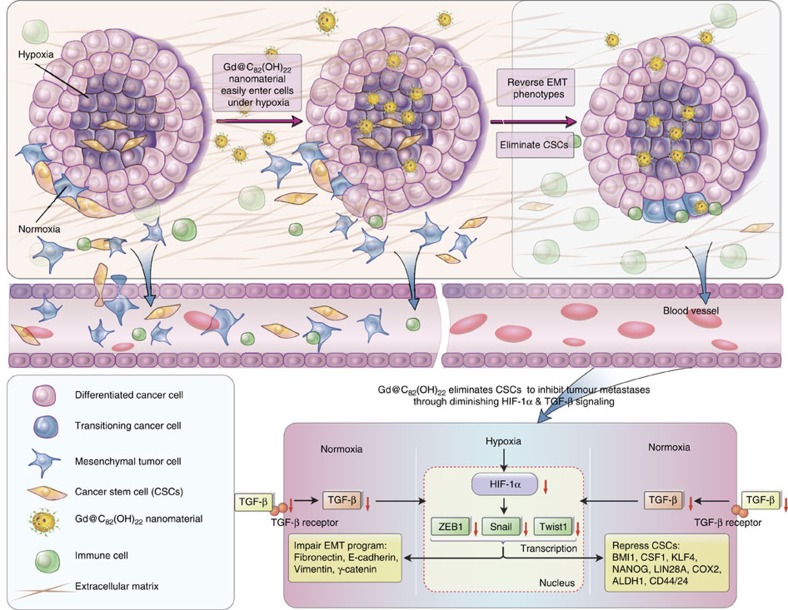
Schematic diagram of key pathways by which Gd@C_82_(OH)_22_ nanoparticles inhibits tumour growth. .

We provide definitive evidence that the Gd@C_82_(OH)_22_ is able to reverse the EMT program of cancer cells and efficiently deplete CSC populations. We suggest the major mechanism by which Gd@C_82_(OH)_22_ targets CSCs may be due to its specificity in abrogating CSC self-renewal and driving terminal differentiation of CSCs, effects not observed in the normal SC-like cells. These studies therefore identify a novel class of nanomaterial-based CSC specific inhibitors with minimal toxicity in normal tissues. Intra-tumoral heterogeneity and intolerable toxicity towards normal tissue are among the leading causes that limit the efficacy of contemporary CSC targeting approaches. Furthermore, toxicity is often even more pronounced with combinatorial strategies employed to increase the efficacy of such therapies^34^,^35^. Interestingly, neither appreciable toxicity towards normal mammary epithelial cells nor systemic somatic toxicity was observed when utilizing Gd@C_82_(OH)_22_ as a CSC inhibitor (Supplementary Fig. 13). Furthermore, the cellular uptake of Gd@C_82_(OH)_22_ is increased under hypoxic conditions in which it abrogates EMT and depletes CSC populations via the simultaneous inhibition of HIF-1α and TGF-β signalling (Fig. 10). The Gd@C_82_(OH)_22_ targeting of HIF-1α and TGF-β signalling may be achieved due to its high efficiency to scavenge reactive oxygen species^22^,^23^, which are known as potent stimulators of HIF-1α and TGF-β expression in cancer cells^36^,^37^,^38^,^39^.

The single cage molecule of a fullerenol has a diameter of <1 nm. When dissolved in aqueous solution, they form nanoparticles as polyanion nano-aggregates, and the nanoparticle size is reduced in more acidic solution^40^,^41^,^42^. It was reported that the smaller sized nanoparticles may allow deeper penetration into tumour tissues^43^. We observed that in the acidic hypoxic condition, the sizes of Gd@C_82_(OH)_22_ nanoparticles are reduced to around 40 nm (Supplementary Fig. 14), which would presumably allow enhanced penetration of the nanoparticles into tumour tissues.

The size variation with pH alterations can be understood by the deprotonation of the hydroxyl groups on the cage surface. It has been previously reported that a decrease in pH occurs when fullerenol powder is added into water^41^. The Gd@C_82_(OH)_22_ cage surface has both attractive (C-OH) and repulsive (C-O^−^) sites. Hence, the acidic protons are involved in attractive hydrogen bonding interactions with other Gd@C_82_(OH)_22_ molecules and this constitutes a driving force of nanoparticle formation^40^. Our measurement of its nanoparticle formation at the different pH solutions indicates that at pH 4.3 solution, the average size of Gd@C_82_(OH)_22_ nanoparticles is ~40 nm, at pH 5.1, ~116 nm and at pH 7.4, ~175 nm. When the pH value is further increased to 8.5, the average size of Gd@C_82_(OH)_22_ nanoparticles decreases to ~84 nm, and at pH 9.7, it is 38 nm, rather smaller (Supplementary Fig. 14).

At pH 4.3, the highly acidic surrounding inhibits the deprotonation of the Gd@C_82_(OH)_22_ surface hydroxyl groups, most hydroxyls present as C-OH with less C-O^−^ formation. Hence, the attractive hydrogen bonding interactions become weak, which thus inhibits the formation of the larger clusters. Compared with the acidic and alkalic surroundings, a neutral condition (at pH 7.4) presents no drive to inhibit the deprotonation process of Gd@C_82_(OH)_22_, it easily produces C-O^−^ groups to form hydrogen bonding with the attractive C-OH groups. Hence, we can understand why the size becomes the largest at the neutral pH value. The alkaline surroundings easily consume the H^+^ dissociated from hydroxyls, so, in the pH 9.7 solution, they mostly present as the repulsive C-O^−^ groups. The repulsive forces among Gd@C_82_(O^−^)_n_ molecules inhibit their aggregation to form larger size nanoparticles.

Conversely, it was reported that the less negatively charged nanoparticle is internalized better by cells^44^,^45^. Hence, we measured the *zeta* potential of Gd@C_82_(OH)_22_ nanoparticles in solutions of different pH values. At pH 4.0~5.0 solutions, ξ (negative value) is only half of that observed at pH 7.0 demonstrating that less negative charges are associated with Gd@C_82_(OH)_22_ nanoparticles in more acidic surroundings, such as would be in a tumour microenvironment. Hence, the physiochemical properties of Gd@C_82_(OH)_22_ may contribute to its selective uptake in areas of hypoxia where it blocks EMT and effectively targets CSC populations. As CSCs contribute to treatment resistance and facilitate tumour metastasis, Gd@C_82_(OH)_22_ and/or related compounds may possess significant clinical utility.

A recent study has reported that treatment with anti-angiogenic agents increased intra-tumoral hypoxia in breast cancer xenografts and resulted in an increase in CSC populations^31^. This increase in CSCs generated by tumour hypoxia was mediated by HIF-1α and may limit the efficacy of antiangiogenic agents. Gd@C_82_(OH)_22_ has also been reported to be a potent inhibitor of tumour angiogenesis^1^,^8^. As Gd@C_82_(OH)_22_ specifically targets CSCs in hypoxia, the use of Gd@C_82_(OH)_22_ might represent a novel strategy to abrogate tumour neo-angiogenesis without exacerbating the CSC population consequent to intra-tumoral hypoxia (Fig. 7a) by effectively targeting both bulk tumour cells and CSCs. Furthermore, the ability of Gd@C_82_(OH)_22_ to accumulate in areas of tumour hypoxia may complement the well-known EPR (Enhanced Permeability and Retention) mechanism by which nanoparticles penetrate and accumulate in tumours via their leaky vasculature^9^,^46^. Thus, the identification of fullerenol nanomaterial with intrinsic CSC specificity represents a novel approach to target this crucial cancer cell population. The apparent absence of significant toxicity of these nanomaterials in normal tissue further highlights their therapeutic potential.

## Methods

### Preparation of Gd@C_82_(OH)_22_ and C_60_(OH)_22_ nanoparticles

Gd@C_82_(OH)_22_ nanoparticles were synthesized by the Krätschmer-Huffman method and extracted by a high-temperature and high-pressure method. Gd@C_82_ was separated and purified using high-performance liquid chromatography (HPLC, LC908-C60, Japan Analytical Industry), and identified by a matrix-assisted laser desorption time-of-flight mass spectrometer (MADLI-TOF-MS, Auto-Flex, Bruker, Germany). Gd@C_82_(OH)_22_ was synthesized by the alkaline reaction and purified by Sephadex G-25 column chromatography (5 × 50 cm) with an eluent of neutralized water.

C_60_(OH)_22_ nanoparticles were prepared. In brief, 2 ml NaOH (2.22 g ml^−1^) and 1 ml tetrabutyl ammonium hydroxide (TBAH) was mixed with 30 ml C_60_ (Sigma Aldrich) toluene (1.5 mg ml^−1^). After the mixture was stirred for 24 h, solution was stirred continuously for another 12 h and then kept motionless for 2 h. The aqueous phase was washed three times. The precipitate was dispersed in ultrapure water after evaporating the methanol. Finally, the dispersion solution of the precipitate was purified using a Sephadex G-25 column (5 × 50 cm) using deionized water as the eluent.

### Cell culture

All cell lines used in the studies were purchased from the American Type Culture Collection (Rockville, Maryland, USA). All cell strains were cryopreserved within three passages and no cell aliquot was cultured continuously for more than 6 months. No cross-contamination of other human cells was observed. The cell lines utilized are 100% matched with those of ATCC. Possible mycoplasma contamination of all cell lines in the laboratory is routinely and regularly monitored using mycoplasma detection set (M&C Gene technology).

MDA-MB-231 and BT549 were cultured in Dulbecco’s modified Eagle’s medium (DMEM) medium supplemented with 10% fetal bovine serum (Gibco), 2 mM L-glutamine, 20 mM HEPES, 100 U ml^−1^ penicillin and 1 mg ml^−1^ streptomycin (Invitrogen). MCF-10A was cultured in DMEM/F12 medium supplemented with 5% horse serum (Invitrogen), 10 μg ml^−1^ insulin, 20 ng ml^−1^ EGF, 100 ng ml^−1^ cholera toxin, 500 ng ml^−1^ hydrocortisone and 1% Antibiotic-Antimycotic (penicillin, streptomycin and amphotericin B).

For normoxic culture, cell lines were cultured in the presence of PBS, Gd@C_82_(OH)_22_, C_60_(OH)_22_ or GdCl_3_ (50 μM) for 21 days at 37 °C in an incubator with 95% air and 5% CO_2_. For hypoxic culture, cells were maintained in stable and controlled hypoxic conditions (1.5% O_2_, 93.5% N_2_, and 5% CO_2_) for 10 days at 37 °C in a modular incubator chamber (Billups-Rothenberg). Partial pressure of oxygen (pO2) was between 7 and 8 kPa in the culture media. For rescue experiments, cells were treated with 20 ng ml^−1^ recombinant human TGF-β (Minneapolis, MN, USA) for 48 h.

### Inductively coupled plasma mass spectrometry analysis

MDA-MB-231 cells were incubated with Gd@C_82_(OH)_22_ nanoparticles (50 μM), and were washed, digested in 1 ml of 1 N HNO_3_. After mixing with 2 ml H_2_O_2_, samples were digested and heated at 150 °C~200 °C in open vessels on a hot plate. The remaining solution (0.5 ml) was cooled to room temperature and diluted to 3 ml with mixed acid solution containing 2% HNO_3_. A blank solution and a series of Gd standard solutions (0.1, 0.5, 1, 5, 10, 50, 100 and 200 p.p.b.) were prepared to obtain a standard curve. Indium (10 p.p.b.) in 2% HNO_3_ was used as an internal standard. Both the standard and the test solutions were measured three times by ICP-MS.

### Cell morphology

Two-dimensional (2D) and three-dimensional (3D) cell culture were used to observe cell morphology. Twenty-four-well plates were pre-coated with growth factor reduced Matrigel. In the 2D culture model, single-cell suspensions were directly plated (2 × 10^4^ cells/well) in complete culture medium on the 100% matrigel bed. In the 3D model, cells (2 × 10^4^ cells/well) were grown in culture wells pre-coated with matrigel and in complete culture medium containing 2% matrigel. After 5 days, all microscopical images were captured using Nikon TE 300 (Nikon, Japan).

Cells were fixed with 2.5% glutaraldehyde overnight and then incubated with 0.1% Triton-100 solution. The actin cytoskeleton in cells was visualized with 300 nM rodamine-labelled Phalloidin (diluted in 1 × PBS) (Invitrogen, USA) for 30 min. Cells were then washed and observed under a confocal microscope with excitation at 561 nm and emission at 615 nm (Perkin Elmer Ultra View Vox system, USA).

### Cell scattering assay

Single cells were seeded sparsely at 1 × 10^3^ cells/100-mm dish in complete culture medium and cultured for 7 days. The number of isolated cell colonies, their size and the degree of scattering were observed by phase-contrast microscopy (Nikon TE 300, Japan). One hundred colonies in each sample were categorized after scoring phase contrast images into three categories: (a) compact (>90% of cells in the colony has cell-cell contacts), (b) loose (50–90% of cells form junctions), (c) scattered (<50% cell form junctions).

### Cell adhesion assay

Cells were washed and resuspended in serum-free media containing 0.02% BSA, and plated at 5 × 10^5^ cells/well in 96-well plates pre-coated with 0.5% gelatin. The cells were incubated for 3 h at 37 °C in 5% CO_2_, and then the plates were washed three times to remove non-adherent cells. Cells were fixed with 2.5% glutaraldehyde and then stained using DAPI (4′, 6-diamidino-2-phenylindole) and photographed.

### Trans-well cell migration and invasion assay

Cell migration and invasion were quantified by trans-well assays using uncoated (8 μm pore size, Corning Costar, USA) or growth factor-reduced Matrigel-coated (8 μm pore size, BD, USA) filters in 24-well plates, respectively. In brief, after treatment with Gd@C_82_(OH)_22_ for 21 days, MDA-MB-231 and BT549 cells were plated in DMEM with 0.2% BSA onto the upper chamber of the trans-wells (2 × 10^4^ cells/well). Below the insert, the chambers of 24-well plates contained DMEM supplemented with 10% FBS. The chambers were incubated at 37 °C with 5% CO_2_ for 6 h (migration assay) or 24 h (invasion assay). At the end of incubation, cells migrating or invading through the filter to the lower surface were fixed with 4% paraformaldehyde for 30 min and stained with 0.1% crystal violet for 10 min. Migrated or invaded cells were photographed and counted in five randomly chosen fields.

### Wound-healing assay

Tumour cell migration was assessed using a wound-healing assay. In six-well plates, cells were cultured until they reached confluent. After culture in serum-free DMEM for 24 h, the monolayers were scratched with woundings by plastic tips and then were gently rinsed with PBS three times. Phase-contrast images were captured after further culture for 0, 12 and 30 h.

### Cell proliferation assay

A cell count kit-8 (CCK-8) (Kumamoto Techno Research Park, Japan) was used to examine cell proliferation. CCK-8 includes WST-8 [2-(2-methoxyl-4-nitrophenyl)-3-(4-nitrophenyl)-5-(2,4- disulfonicacid benzene)-2H-tetrazalium sodium] that can be reduced to highly water-soluble formazan dye, which is yellow. In brief, cells were incubated with 100 μl of culture medium in 96-multiwell plates. Media were removed and 100 μl DMEM containing CCK-8 (10%) was added to each well. After 2 h incubation at 37 °C, the absorbance at 450 nm of each well was measured using a standard enzyme-linked immunosorbent assay (ELISA)-format spectrophotometer.

### Detection of apoptosis and necrosis

Apoptotic cells and necrotic cells were analyzed by double staining with Alexa Fluor 488 annexin V and propidium iodide (PI) (Invitrogen, USA), in which Alexa Fluor 488 annexin V bound to apoptotic cells with exposed phosphatidylserines (PS), while PI labelled necrotic cells with membrane damage. All cells (floating and adherent) were collected and washed once with cold PBS. Five microitres Alexa Fluor 488 Annexin V was added to the cell suspension in the presence of 100 μl 1 × binding buffer and incubated for 20 min at room temperature. Cells were co-stained with 100 μl PI (1 μg ml^−1^) and immediately analysed or sorted using BD flow cytometer (BD, USA). The percentage of apoptotic (annexin+/PI−) and necrotic (annexin+/PI+) cells was determined using software. Data represent the mean fluorescence obtained from a population of 10,000 cells.

### ELISA analysis of TGF-β, IL-6 and IL-8 levels

Levels of TGF-β, IL-6 and IL-8 in culture supernatants for MDA-MB-231 cells were determined by a specific ELISA according to the manufacturer’s instructions, using matched antibody pairs and recombinant cytokines as standards (Cell Signaling Technology, USA). In brief, cells were cultured with PBS, Gd@C_82_(OH)_22_, C_60_(OH)_22_ or GdCl_3_ for 3, 7, 14, 21 and 28 days. Cells were washed and fresh media was added. After 24 h, culture supernatants were collected. Ninety-six-multiwell plates coated with the corresponding purified anti-human capture monoclonal antibody were used. Culture supernatants and serial dilutions of the standard were added to each well and incubated for 90 min at 37 °C. After four washes, bound samples were detected using the corresponding biotinylated anti-mouse antibody at 37 °C for 1 h. After another four washes, avidin-horseradish peroxidase solution was added, and plates were incubated at 37 °C for 30 min. After the final four washes, plates were kept at 37 °C for 20 min to react with the substrate solution. A 100 ml of blocking solution was added to stop the reaction, and the absorbance at 450 nm was then recorded. Results were expressed in pg ml^−1^, and three independent experiments were performed.

### RNA extraction and quantitative real-time PCR

Total RNA was extracted from cultured cell lines and primary tumours using Trizol reagent following the manufacturer’s guideline (Invitrogen, USA). mRNA was converted to cDNA using SuperScript III Reverse Transcriptase (Invitrogen, USA), and then expression levels of analysed genes were determined using the SYBR Premix Ex Taq Kit (Takara, Japan). The relative amount of gene transcripts was normalized to *GAPDH*. Primers are listed in Supplementary Table 5.

### Western blotting

Monolayer cells were washed three times using ice-cold PBS and lysed using RIPA lysis buffer (40 mM Tris, 150 mM NaCl, 10 mM ethylenediamine tetraacetic acid, 10% glycerol, 1% Triton X-100, 10 mM glycerophosphate, 1 mM Na_3_VO_4_, 1 mM phenylmethylsulfonyl fluoride) supplemented with complete protease inhibitor cocktail tablets (Roche, Switzerland). BCA protein assay (Thermo Fisher Scientific, Australia) was used to determine protein concentration. Fifty micrograms of proteins were separated by SDS–polyacrylamide gel electrophoresis (SDS–PAGE) and transferred onto a polyvinylidene difluoride membrane (Bio-Rad, USA). After blocking for 60 min with 2% BSA, membranes were incubated with the primary antibody overnight at 4 °C followed by incubation with the corresponding secondary antibody for 60 min at room temperature. The primary antibodies were used (Supplementary Table 6): E-CADHERIN, γ-CATENIN, VIMENTIN and FIBRONECTIN-1, β-ACTIN, E47, TWIST1, TGF-β, HIF-1α, HIF-1β, SNAIL, ZEB1 and COX-2. Membranes were visualized with an ECL-plus detection system (GE Healthcare, NJ) after incubation with an anti-mouse or rabbit secondary antibody (GE healthcare). Images have been cropped for presentation. Full-size images are presented in Supplementary Fig. 16.

### Transient transfection

Human pcDNA3.1/HIF-1α was constructed and purified using the EndoFree Plasmid Maxi kit (QIAGEN, USA) for transfection. Human breast cancer cell MDA-MB-231 was transiently transfected with a pcDNA3.1/HIF-1α (Addgene plasmid 18949), or empty vector (pcDNA3.1) as control using Lipofectamine 2000 and Plus reagent in six-well plates. The transfections were performed according to the manufacturer’s instructions. After transfection, cells with various treatments were collected for the following experiments.

### Tumoursphere culture

For tumoursphere culture *in vitro*, cells (5 × 10^4^ cells ml^−1^) were seeded in ultra-low attachment dishes (Corning, USA) and grown in serum-free DMEM/F12 medium (Lonza) supplemented with hormone mixture B27 (Gibco, USA), 20 ng ml^−1^ EGF (Sigma-Aldrich, USA), 10 ng ml^−1^ bFGF (Invitrogen, USA) and 1 μg ml^−1^ heparin. Fresh medium was added to the culture every 48 or 72 h. Cells were incubated in a 5% CO_2_ incubator at 37 °C for 7–14 days, and tumourspheres were counted under microscope with size of 70–150 μm and >150 μm.

Tumour tissues were isolated from PBS, Gd@C_82_(OH)_22_, C_60_(OH)_22_ or GdCl_3_-treated mice and mechanically dissociated using sterile scalpels into pieces followed by enzymatic dissociation in collagenase (300 U ml^−1^) and hyaluronidase (100 U ml^−1^) (Stem Cell Technologies) diluted in complete DMEM/F12 media for 3–5 h at 37 °C with agitation every 20–25 min. The cell suspension was sequentially filtered and centrifuged at 300 × *g* for 10 min. The cell pellet was resuspended in complete DMEM/F12 media and cultured in ultra-low attachment dishes. Cells were incubated in a 5% CO_2_ incubator at 37 °C for 7–14 days, and tumourspheres were counted under microscope with size of 70–150 μm and >150 μm.

### Animal models

Four- to six-week-old female BALB/c nude mice with body weights in the range of 15.0 to 17.0 g were used as hosts for tumour xenografts. They were housed in a temperature-controlled, ventilated and standardized disinfected animal room. Mice were allowed to acclimatize, without handling, for a minimum of 1 week before the start of experiments. All animal experiments were conducted using protocols approved by the Institutional Animal Care and Use Committee at the Institute of Tumors of the Chinese Academy of Medical Sciences.

Two hundred mice were divided randomly into 36 groups according to the Supplementary Table 4. **Groups A, B, C, D** and **E** stand for mice models for detecting the tumour initialing capacity of cancer stem cells. Different numbers (5 × 10^6^, 5 × 10^5^, 5 × 10^4^, 5 × 10^3^, 5 × 10^2^) of MDA-MB-231 cells were injected into the right back flanks of the mice *s.c.* Mice were monitored and treated with PBS, Gd@C_82_(OH)_22_, C_60_(OH)_22_ or GdCl_3_ daily^47^,^48^,^49^,^50^,^51^,^52^. **Group F** is referred as approach I (early treatment) model. A total of 1 × 10^6^ MDA-MB-231 cells were injected and mice were treated at day 0 for 21 days. **Group G** is referred as approach II (terminal treatment) model. A total of 1 × 10^6^ MDA-MB-231 cells were injected. When the tumours grew to 100 mm^3^, the mice were treated for 21 days^47^,^48^. **Group H** is referred as an agent withdrawal model. A total of 1 × 10^6^ MDA-MB-231 cells were injected and mice were treated at day 0 for 21 days. From the 22nd day, mice were not offered any administration before killing at day 29. **Group I** is the metastasis model with tumour cell tail vein injection (*i.v*). A total of 1 × 10^6^ MDA-MB-231 cells were injected i.v. and mice were treated at day 0 for 21 days^47^,^48^.

Cells were washed twice with cold PBS. Immediately before injection, cells were combined with Matrigel (BD Biosciences) at a 1:1 ratio. Cell suspensions were injected into the right back flanks of the mice by s.c. inoculation. Tumour-bearing mice were administered with either PBS, or 2.5 μmol kg^−1^ Gd@C_82_(OH)_22_, C_60_(OH)_22_ or GdCl_3_, once a day. Tumour size was measured using a caliper every 2 or 3 days. Tumour volumes were calculated according to the following formula: tumour volume (mm^3^)=1/2 × a × b × b (where ‘a’ is vertical long diameter and ‘b’ is vertical short diameter). Mice were killed and tumours were weighed. Tumour inhibition rates were calculated using the following formula: rate of inhibition (%)=(mean tumour weight of untreated PBS control−mean tumour weight of treated group)/mean tumour weight of untreated PBS control × 100. Pulmonary metastasis was analysed macroscopically and by H&E tissue staining.

### Haematoxylin and eosin staining analysis of tumours and organs

Immediately after surgical removal, tumours, lungs and livers were fixed overnight in 10% formalin neutral buffer, dehydrated in a series of graded ethanol solutions and embedded in paraffin. Baseline histological slides containing sections (4–5 μm in thickness) were stained with haematoxylin/Eosin (HE) followed by dehydration through a graded series of ethanol solutions from 75 to 100%, and examined blindly by a well-trained pathologist. Histological observations and photomicrography were performed using a light microscope (Nikon U-III multipoint sensor system). An average of five fields of view ( × 100) was used to calculate the number of metastases.

### Immunohistochemistry from primary tumour sections

For IHC, sections were rehydrated followed by heat-induced epitope retrieval and incubated for 10 min with 0.3% H_2_O_2_ in water to block endogenous peroxidase. Sections were incubated with blocking buffer (10% normal goat serum diluted in PBS) for 60 min at room temperature and then with a primary antibody diluted in blocking buffer overnight at 4 °C. Primary monoclonal antibodies used were E-CADHERIN, γ-CATENIN, VIMENTIN and FIBRONECTIN-1 (Supplementary Table 6). The following day, the biotinylated secondary antibody was used to incubate the sections for 60 min at room temperature. And then, streptavidin/biotin HRP-conjugate was added to the sections for 30 min. The signal was developed using the Vector DAB substrate kit according to the manufacturers’ instructions.

### Immunofluorescence

For immunofluorescence, cells were cultured on coverslips and fixed with 4% paraformaldehyde in PBS for 20 min at room temperature. After permeabilization with 0.1% Triton X-100 in PBS for 5 min, cells were blocked with 1% BSA for 1 h. Primary monoclonal antibodies used included E-CADHERIN, γ-CATENIN, VIMENTIN and FIBRONECTIN-1 (Supplementary Table 6). Visualizaton was achieved with the secondary antibodies conjugated with 488 Alexa dye were applied and DAPI (Biotium, USA) was used for nuclear staining. Slides were mounted with Slow Fade (Invitrogen) and kept at 4 °C.

### Statistical analysis

Values are shown as the mean values±s.e.m. The numbers of samples or mice per group used in each experiment are indicated in the corresponding figure legends as *n*. All experiments shown were performed at least three independent times. All the statistical analyses were generated using GraphPad Prism 5 (GraphPad Software). Normally distributed data were analysed using one-way or two-way analysis of variance (ANOVA) for multiple comparisons. One-way ANOVA analyses were followed by Tukey’s *post-hoc* tests, whereas two-way ANOVA analyses were followed by Bonferroni’s *post-hoc* tests. The incidence of tumours was analysed using the χ^2^-test. The statistical test used and *P* values are indicated in each figure legend. *P*<0.05 was considered to indicate statistical significance. *P<0.05, ^#^*P*<0.05 or ^&^*P*<0.05, **P<0.01, ^##^*P*<0.01 or ^&&^*P*<0.05.

## Author contributions

Y.L., P.Q. and C.C designed and performed the experiments, collected and analysed the data and co-wrote the manuscript. Y.Z. and T.Z. conceived the principal idea, designed the experiments and wrote the manuscript. B.S. and J.D. provided Gd@C_82_(OH)_22_ nanoparticles. X.L., X.Z. and W.Z. performed the qPCR and western blot experiments. H.L., Z.C., L.W., R.B. and J.T. performed animal experiments. X.G. ran the density functional theory calculations. H.Z., Q.W. M.S.W., P.E.L. and S.L. interpreted the data and provided advice. All the authors discussed the results and commented on the manuscript.

## Additional information

**How to cite this article:** Liu, Y. *et al*. Gd-metallofullerenol nanomaterial as non-toxic breast cancer stem cell-specific inhibitor. *Nat. Commun.* 6:5988 doi: 10.1038/ncomms6988 (2015).

## Supplementary Material

Supplementary Information Supplementary Figures 1-16 and Supplementary Tables 1-6

## Figures and Tables

**Table 1:** The effect of Gd@C_82_(OH)_22_ treatments on MDA-MB-231 *in vivo* tumorigenesis.

**Tumour/injected cell number**	**5 × 10**^**6**^	**5 × 10**^**5**^	**5 × 10**^**4**^	**5 × 10**^**3**^	**5 × 10**^**2**^
PBS*	6/6	5/6	5/6	4/6	1/6
**Gd@C**_**82**_**(OH)**_22_*	6/6	5/6	2/6	0/6	0/6
**C**_**60**_**(OH)**_22_*	6/6	5/6	3/6	3/6	2/6
**GdCl**_3_*	6/6	6/6	5/6	3/6	1/6

^*^Different numbers of MDA-MB-231 cells were injected s.c. and mice were treated with PBS, Gd@C_82_(OH)_22_, C_60_(OH)_22_ or GdCl_3_ daily. The numbers represent ‘the number of mice with tumorigenesis/the number of mice in every group’.
