# Supplementary material for: Gd-metallofullerenol nanomaterial as non-toxic breast cancer stem cell-specific inhibitor
Source: Nat Commun. 2015 Jan 23;6:5988. doi: 10.1038/ncomms6988 (PMC4354030; doi:10.1038/ncomms6988)
Supplement: Supplementary Information — Supplementary Figures 1-16 and Supplementary Tables 1-6 [file ncomms6988-s1.pdf]

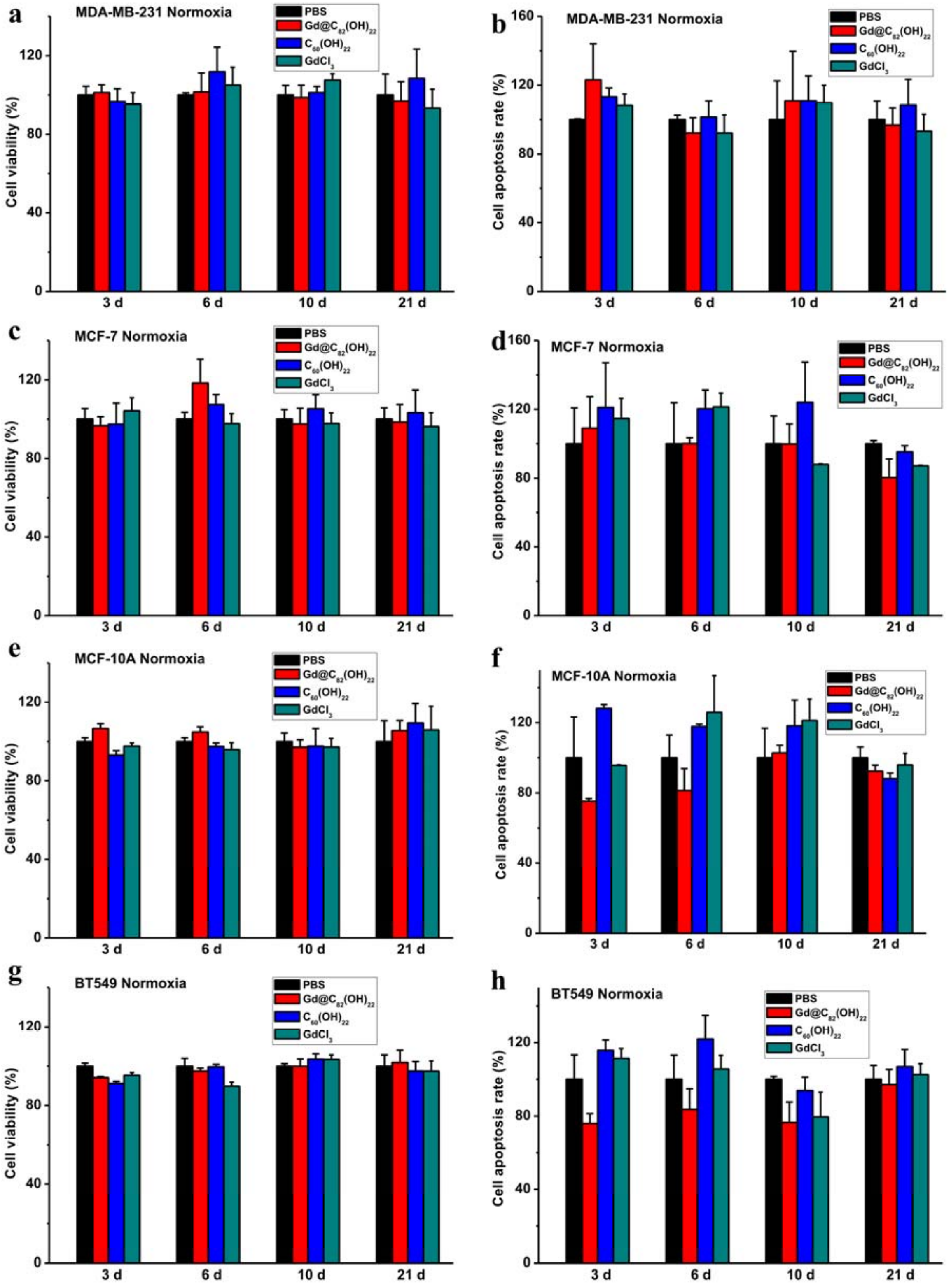

Supplementary Figure 1. Gd@$C_{82}(OH)_{22}$ nanoparticles did not affect cell viability and apoposis. MDA-MB-231, MCF-7, MCF-10A and BT549 cells were treated with PBS, Gd@$C_{82}(OH)_{22}$, $C_{60}(OH)_{22}$ or $GdCl_3$ (all 50 μM) for 3, 6, 10 or 21 days. (a, c, e, g) Cell viability was detected using CCK-8 assay. (b, d, f, h) Cells apoptosis analysis was detected using double staining with annexin V and PI. All the data are represented as mean ± s.e.m. (n = 3 each).

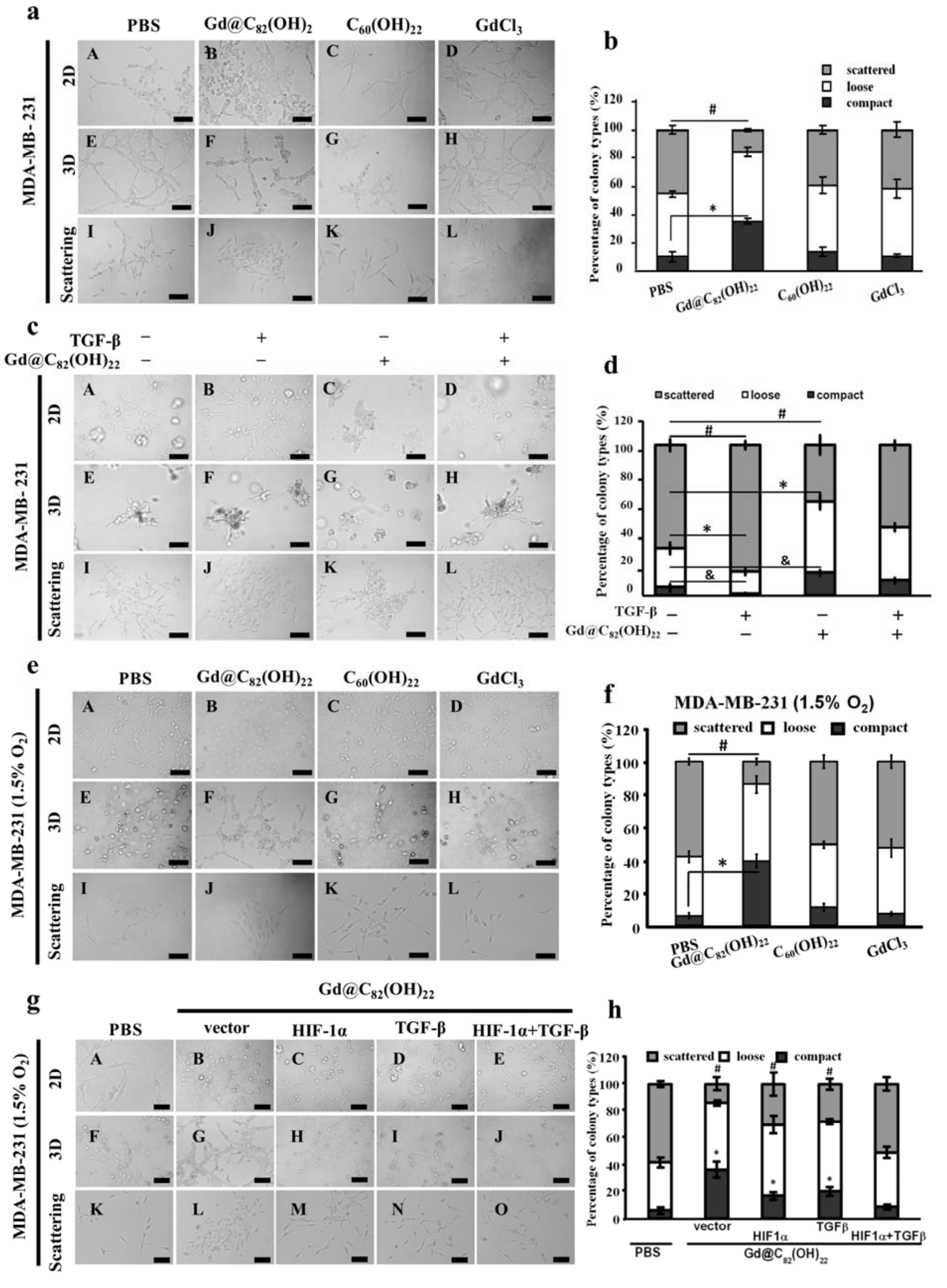

Supplementary Figure 2. MDA-MB-231 cells were treated with PBS (A, E, I), Gd@$C_{82}(OH)_{22}$ (B, F, J), $C_{60}(OH)_{22}$ (C, G, K) or $GdCl_3$ (D, H, L) (50 μM) for 21 days. Cellular morphology of cells cultured on 2D/3D-matrigel cultures (a) and Scattering assay (b) were visualized. mean ± s.e.m. (n = 3 each). To compact cells,

*$p < 0.05$; to scattered cells, $^{\#}p < 0.05$ (one-way ANOVA, Tukey's post-hoc test). MDA-MB-231 cells were cultured with 20 ng/ml TGF-β supplement for 24 hours after treatment with PBS or Gd@$C_{82}(OH)_{22}$ for 21 days and further seeded for 2D- and 3D-matrigel cultures. Morphological changes (c) and Scattering assay (d) were observed. Scale bar = 50 μm. mean ± s.e.m. (n = 3 each). To loose cells, *$p < 0.05$; to scattered cells, $^{\#}p < 0.05$; to compact cells, $^{\&}p < 0.05$ (two-way ANOVA, Bonferroni's post-hoc test). MDA-MB-231 cells were cultured in hypoxia and treated with PBS (A, E, I), 50 μM Gd@$C_{82}(OH)_{22}$ (B, F, J), $C_{60}(OH)_{22}$ (C, G, K) and $GdCl_3$ (D, H, L) for 10 days. Cellular morphology of cells cultured on 2D/3D-matrigel cultures (Scale bar = 50 μm) (e) and Scattering assay (f) were visualization. mean ± s.e.m. (n = 3 each). To compact cells, *$p < 0.05$; to scattered cells, $^{\#}p < 0.05$ (one-way ANOVA, Tukey's post-hoc test). MDA-MB-231 cells were transfected with HIF-1α expressing plasmid and/or treated with 20 ng/ml TGF-β with further culture in presence of Gd@$C_{82}(OH)_{22}$ or PBS under hypoxia for 10 days. Cellular morphology of cells cultured on 2D/3D-matrigel cultures (Scale bar = 50 μm) (g) and Scattering assay (h) were visualized. mean ± s.e.m. (n = 3 each). To compact cells, *$p < 0.05$; to scattered cells, $^{\#}p < 0.05$ (two-way ANOVA, Bonferroni's post-hoc test).

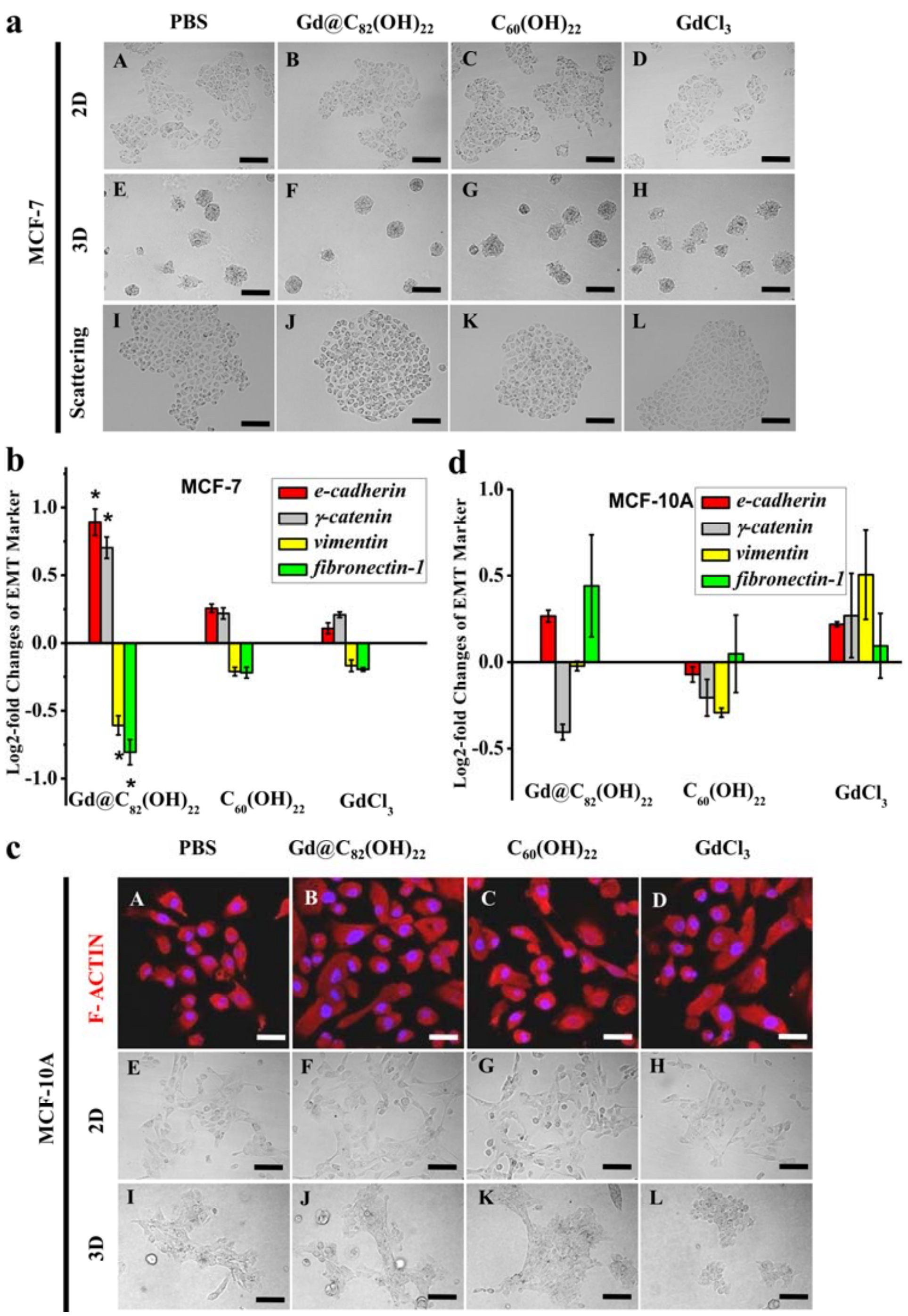

Supplementary Figure 3. MCF-7 and MCF-10A cells were treated with PBS, Gd@$C_{82}(OH)_{22}$, $C_{60}(OH)_{22}$ and $GdCl_3$ (all 50 μM) for 21 days. (a) Cellular morphology of MCF-7 cells was observed on 2D- and 3D-matrigel culture or monolayer adherent culture. Scale bar = 50 μm. (c) Cellular morphology of MCF-10A cells on 2D- and 3D-matrigel cultures. Scale bar = 50 μm. Actin cytoskeleton (red) was observed. (Scale bar = 12.5 μm). (b, d) mRNA levels of EMT markers (*e-cadherin*, *γ-catenin*, *vimentin* and *fibronectin-1*) were analyzed by real-time PCR (mean ± s.e.m., n = 3 each). *$p < 0.05$ (one-way ANOVA, Tukey's post-hoc test).

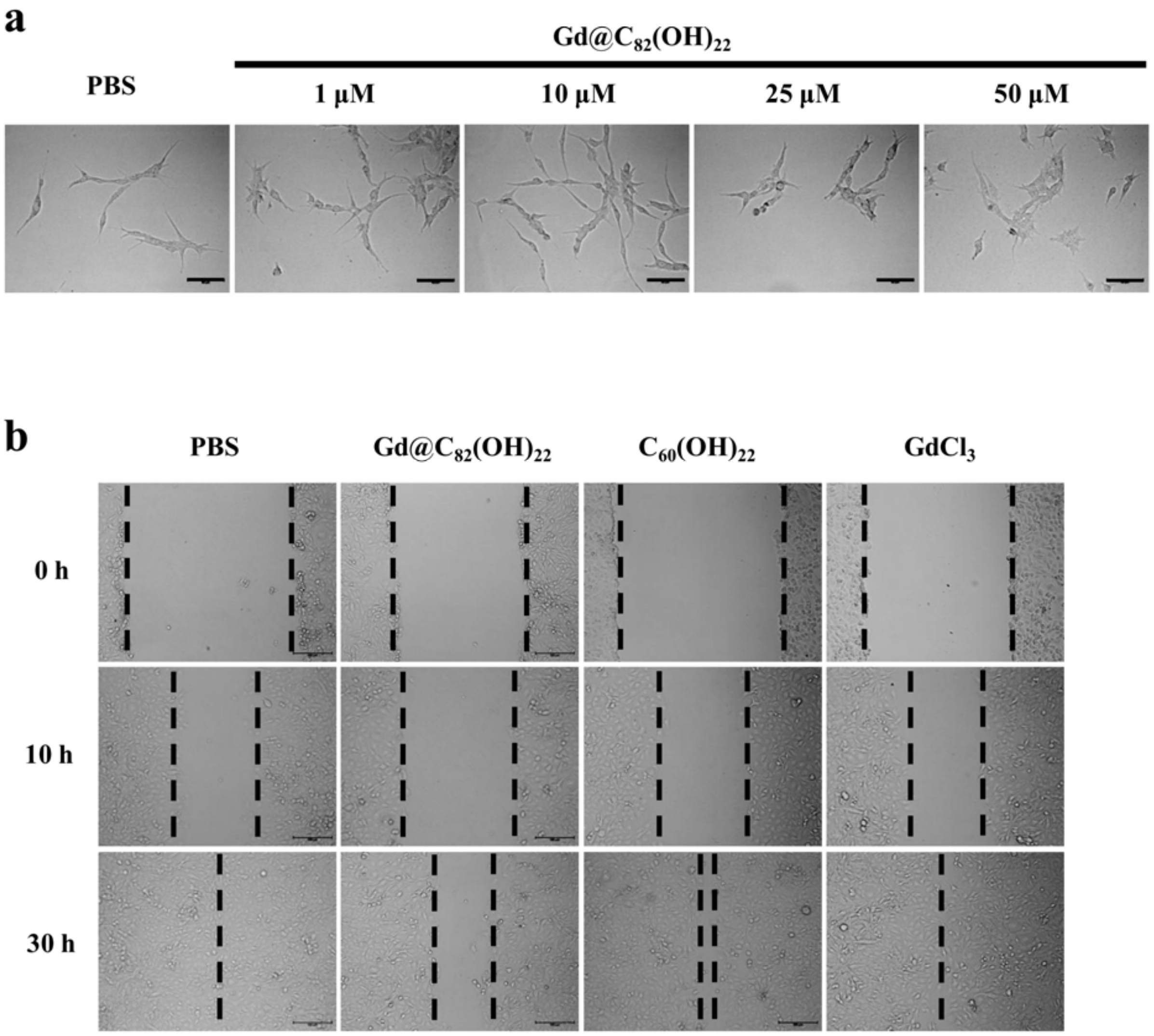

Supplementary Figure 4. (a) MDA-MB-231 cells were cultured and treated for 21 days with PBS and Gd@$C_{82}(OH)_{22}$ (0.1, 1, 10 and 50 μM). Representative images are shown. (b) Cell motility was evaluated by wound healing assay.

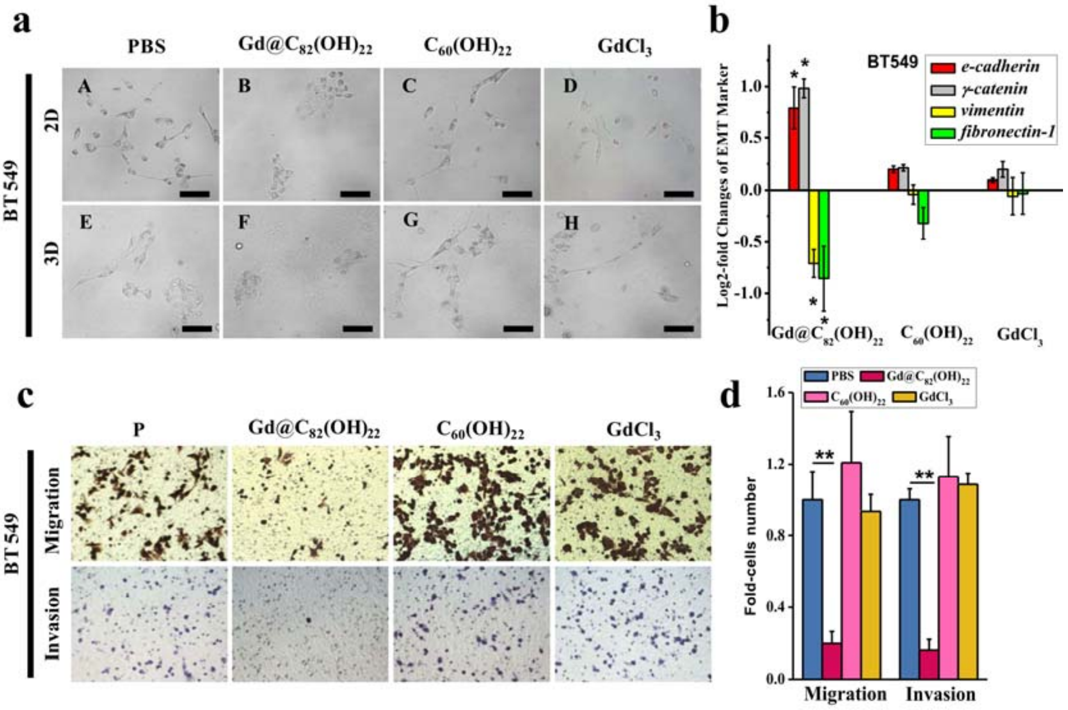

Supplementary Figure 5. BT549 cells were treated with PBS, Gd@$C_{82}(OH)_{22}$, $C_{60}(OH)_{22}$ or $GdCl_3$ (50 μM) for 21 days. (a) Cellular morphology was observed in 2D- and 3D-matrigel cultures. Scale bar = 50 μm. (b) mRNA levels of EMT markers (*e-cadherin*, *γ-catenin*, *vimentin* and *fibronectin-1*) were analyzed by real-time PCR (mean ± s.e.m., n = 3 each). **p* < 0.05 (one-way ANOVA, Tukey's post-hoc test). (c, d) Cell migration and invasion were examined using trans-well cell culture chambers and Matrigel-coated ones (mean ± s.e.m., n = 6 each). **p* < 0.05 and ***p* < 0.01 (one-way ANOVA, Tukey's post-hoc test).

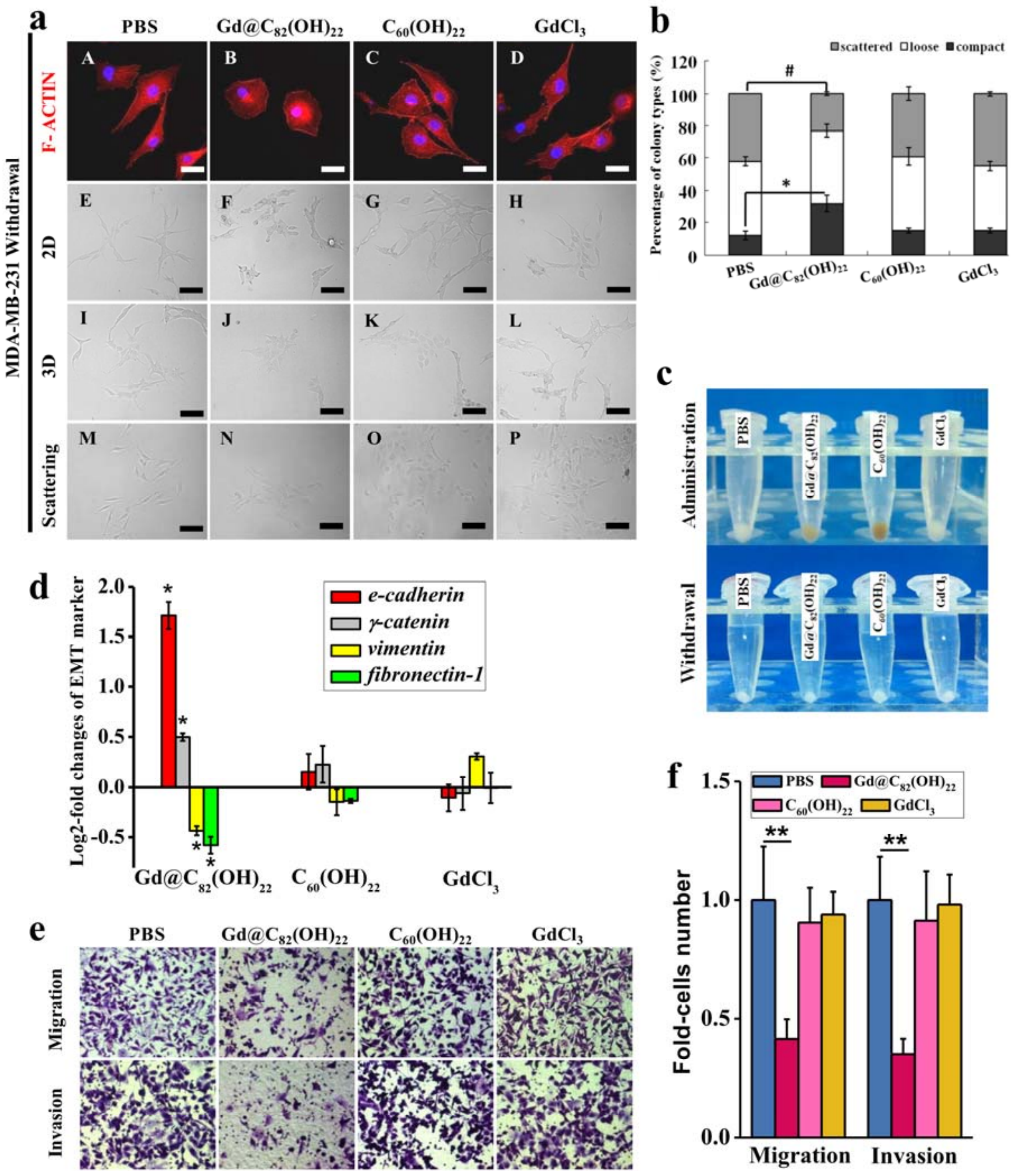

Supplementary Figure 6. MDA-MB-231 cells were treated with PBS, Gd@$C_{82}(OH)_{22}$, $C_{60}(OH)_{22}$ or $GdCl_3$ for 21 days and then subjected to agent withdrawal of for 14-day. (a) Cellular morphology. Scale bar = 50 μm. Actin cytoskeleton (red) was observed. (Scale bar = 12.5 μm). (b) Cell scattering assay. (mean ± s.e.m., n = 3 each). To compact cells, *$p < 0.05$; to scattered cells, $^{\#}p < 0.05$ (one-way ANOVA, Tukey's post-hoc test). (c) The changes of color of cell mass prepared by centrifugation before and after withdrawal. (d) The mRNA level of EMT markers (*e-cadherin*, *γ-catenin*, *vimentin* and *fibronectin-1*) were analyzed by real-time PCR (mean ± s.e.m., n = 3 each). *$p < 0.05$ (one-way ANOVA, Tukey's post-hoc test). (e, f) Cell migration and invasion were examined using trans-well cell culture chambers and Matrigel-coated ones (mean ± s.e.m., n = 6 each). **$p < 0.01$ (one-way ANOVA, Tukey's post-hoc test).

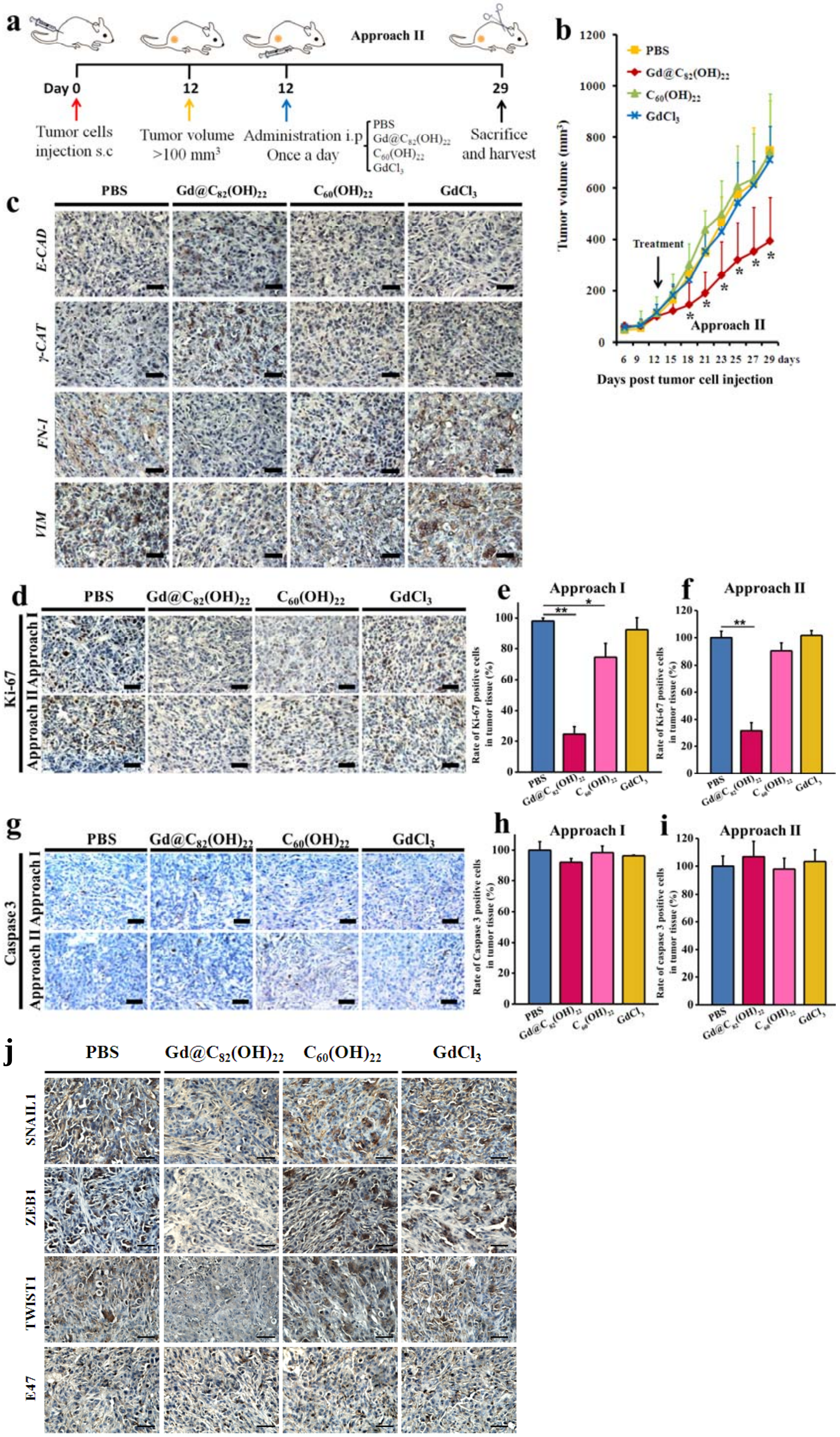
a
Approach II
Day 0
12
12
29
Tumor cells injection s.c
Tumor volume >100 mm³
Administration i.p Once a day
PBS
Gd@C82(OH)22
C60(OH)22
GdCl3
Sacrifice and harvest
b
PBS
Gd@C82(OH)22
C60(OH)22
GdCl3
Tumor volume (mm³)
Treatment
Approach II
Days post tumor cell injection
c
E-CAD
γ-CAT
FN-1
VIM
d
Ki-67
Approach I
Approach II
e
Approach I
Rate of Ki-67 positive cells in tumor tissue (%)
f
Approach II
g
Caspase 3
h
Approach I
Rate of Caspase 3 positive cells in tumor tissue (%)
i
Approach II
j
SNAIL1
ZEB1
TWIST1
E47

Supplementary Figure 7. Inhibition of tumor growth by Gd@$C_{82}(OH)_{22}$ in a mouse xenograft model. (a) Experimental design of approach II (terminal treatment) mice model. Tumor growth curves in approach II (b) mice were plotted. (mean ± s.e.m., n = 5 each). *$p < 0.05$ (two-way ANOVA, Bonferroni's post-hoc test). (c) Expressions of EMT markers in tumors (approach I) sections were detected using immuno-histochemical method (Scale bars represent 25 μm). Expression of Ki-67 (d) and active-caspase-3 (g) in tumor tissues of the approach I or II mice (Scale bars represent 25 μm). Ki-67 (e, f) and active-caspase-3 (h, i) positive cells were quantified (mean ± s.e.m., n = 3 each). *$p < 0.05$ and **$p < 0.01$ (one-way ANOVA, Tukey's post-hoc test). (j) Expression of SNAIL, ZEB1, TWIST1 and E47 in tumors were detected using immuno-histochemical method.

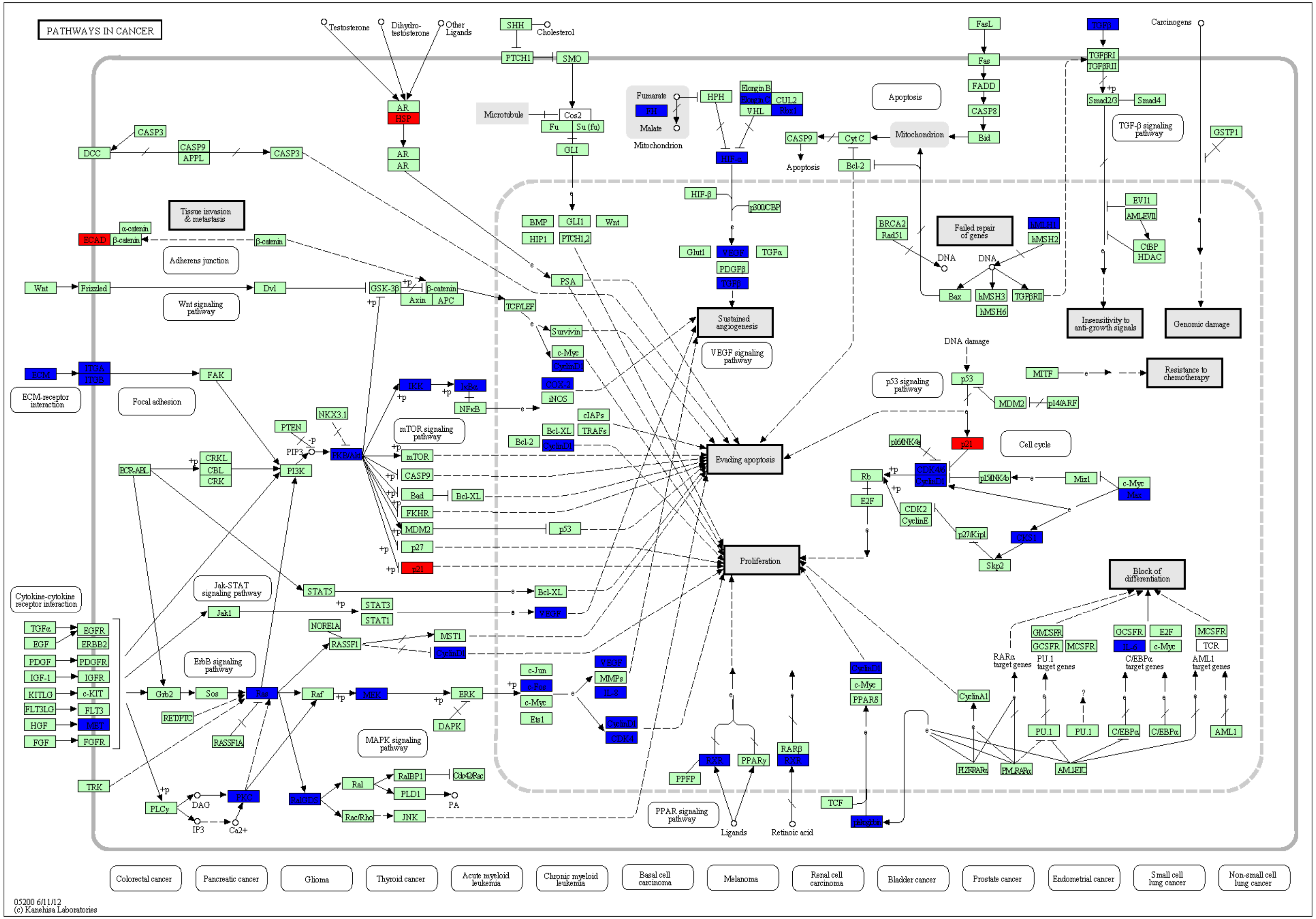

Supplementary Figure 8. KEGG (Kyoto Encyclopedia of Genes and Genomes) analysis of cancer pathway in MDA-MB-231 cells treated with PBS or Gd@$C_{82}(OH)_{22}$. Nodes were labeled using the gene names. Both the diminished (blue) and elevated (red) gene expression are indicated.

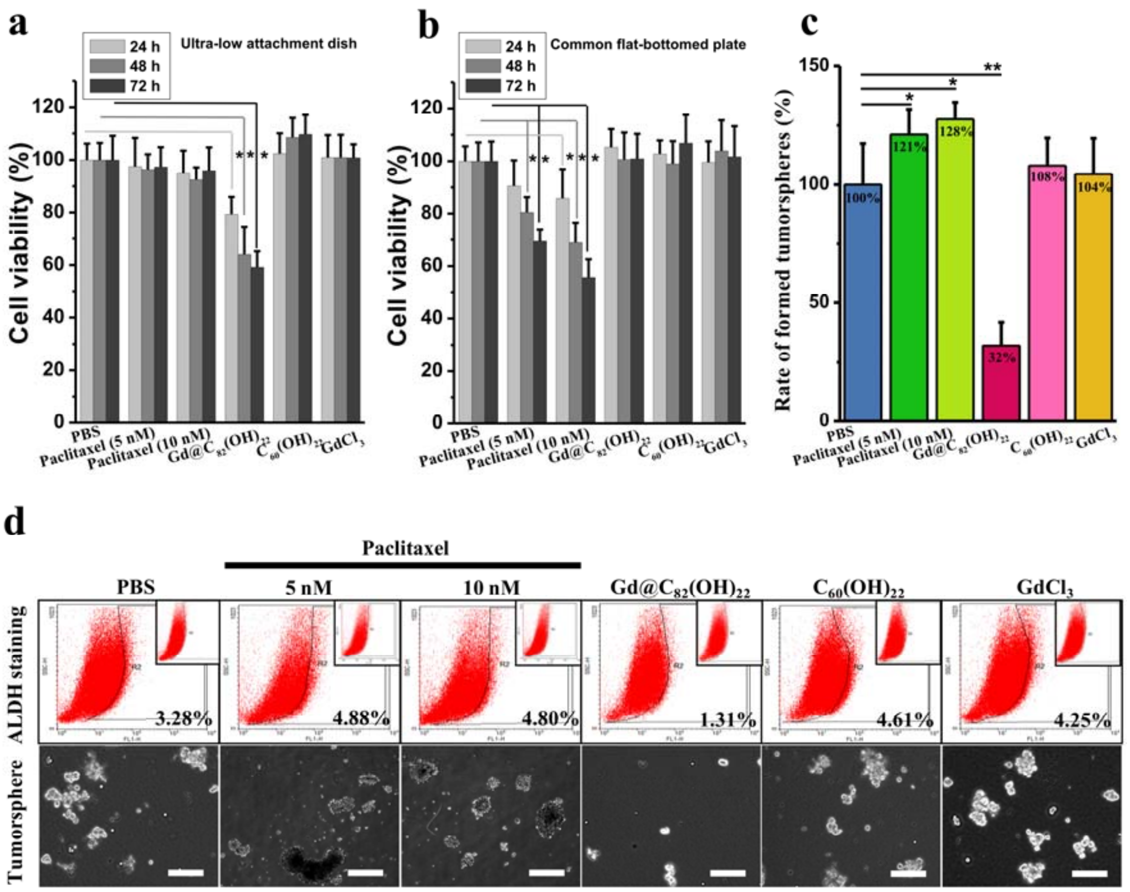

Supplementary Figure 9. BT549 cells were cultured in ultra-low attachment dishes (a) or adherent monolayer culture plate (b) for 10 days, and treated with PBS, Paclitaxel (5 nM or 10 nM), Gd@$C_{82}(OH)_{22}$, $C_{60}(OH)_{22}$ or $GdCl_3$ (50 μM) for another 24 h, 48 h or 72 h. Cell viability was evaluated using CCK-8 assay. After treatment with PBS, Paclitaxel (5 nM or 10 nM), Gd@$C_{82}(OH)_{22}$, $C_{60}(OH)_{22}$ or $GdCl_3$ (50 μM) for 21 days, BT549 were dispersed into single cells and plated in suspension cultures. Tumorspheres were evaluated using the ALDEFLUOR assay (c) and quantified (d). All the data are represented as mean ± s.e.m. (n = 3 each) with *$p < 0.05$ and **$p < 0.01$ (one-way ANOVA, Tukey's post-hoc test).

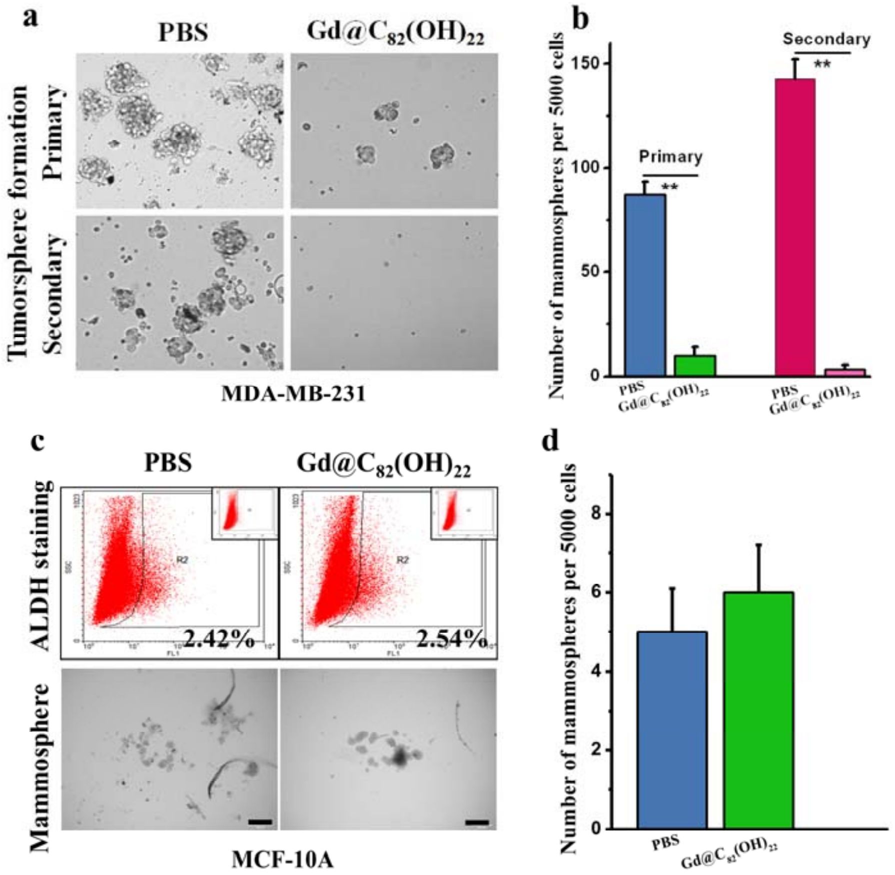

Supplementary Figure 10. $Gd@C_{82}(OH)_{22}$ significantly inhibited the formation of primary and secondary tumorsphere formation and did not appreciably inhibit normal epithelial stem cells. MDA-MB-231 cells were maintained in PBS and $Gd@C_{82}(OH)_{22}$ for 21 days and further dispersed into single cells for mammosphere formation assay. The primary mammospheres were dispersed into single cells for secondary mammosphere formation assay as a measure of self-renewal. a) Primary and secondary mammosphere. b) The tumorspheres (> 70 μm) were quantitated (mean ± s.e.m., n = 3 each). $^{**}p < 0.01$ (one-way ANOVA, Tukey's post-hoc test). MCF-10A cells were maintained in PBS and $Gd@C_{82}(OH)_{22}$ for 21 days and further dispersed into single cells for ALDEFLUOR assay and mammosphere formation assay (c). The mammopheres were counted (d) by microscopy (> 70 μm) (mean ± s.e.m., n = 3 each).

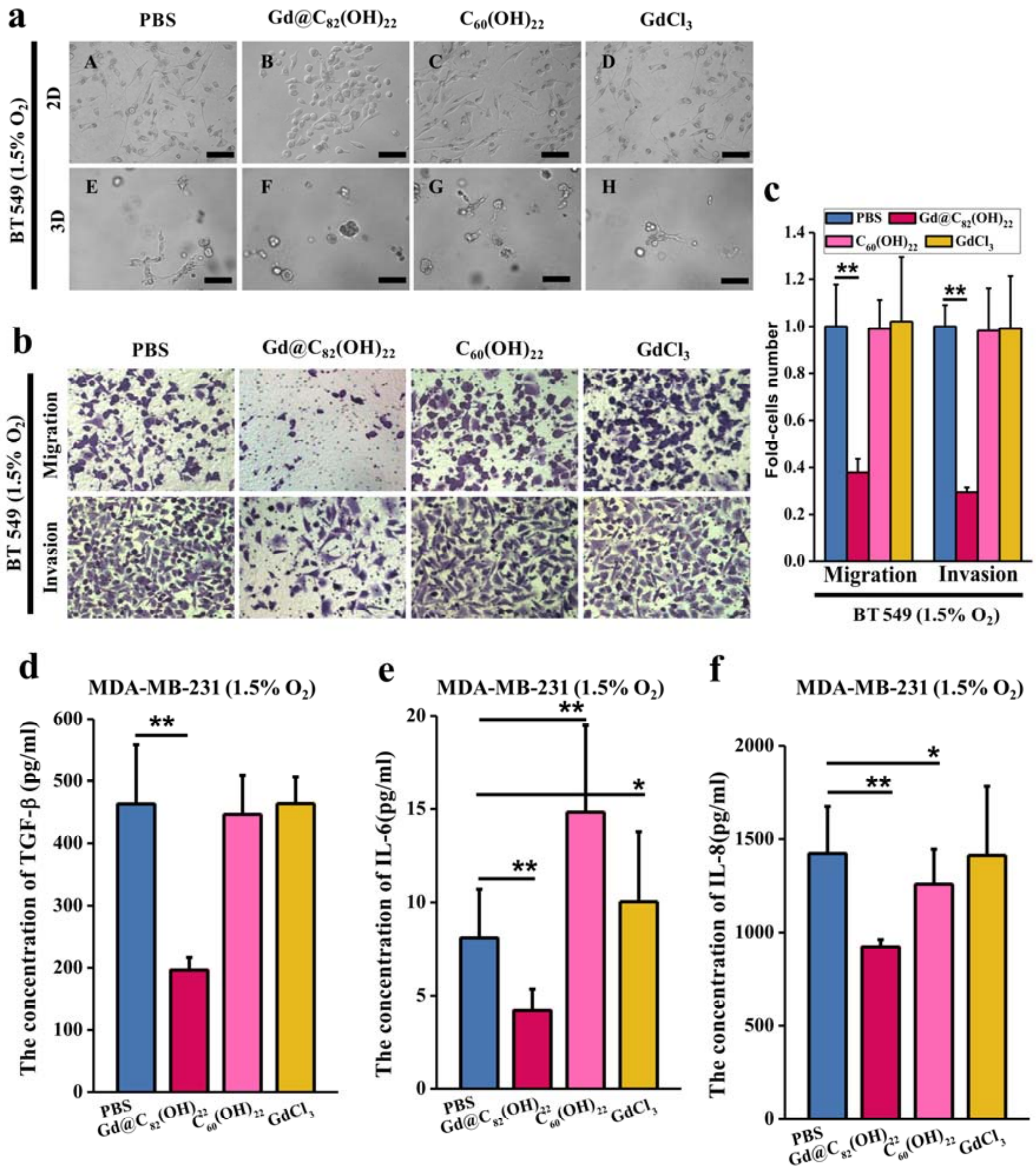

Supplementary Figure 11. BT549 cells were treated with PBS, Gd@$C_{82}(OH)_{22}$, $C_{60}(OH)_{22}$ and $GdCl_3$ (all 50 μM) under hypoxia for 10 days. (a) Cellular morphology on 2D- and 3D-matrigel cultures. (b, c) Cell migration and invasion were examined using trans-well cell culture chambers and Matrigel-coated ones (mean ± s.e.m., n = 3 each). **p < 0.01 (one-way ANOVA, Tukey's post-hoc test). MDA-MB-231 cells were treated with PBS, Gd@$C_{82}(OH)_{22}$, $C_{60}(OH)_{22}$ or $GdCl_3$ (50 μM) for 10 days under hypoxia. ELISA analysis for expression of TGF-β (d), IL-6 (e) and IL-8 (f) in supernatant culture medium were determined. All the data are represented as mean ± s.e.m. (n = 3 each) with *p < 0.05 and **p < 0.01 (one-way ANOVA, Tukey's post-hoc test).

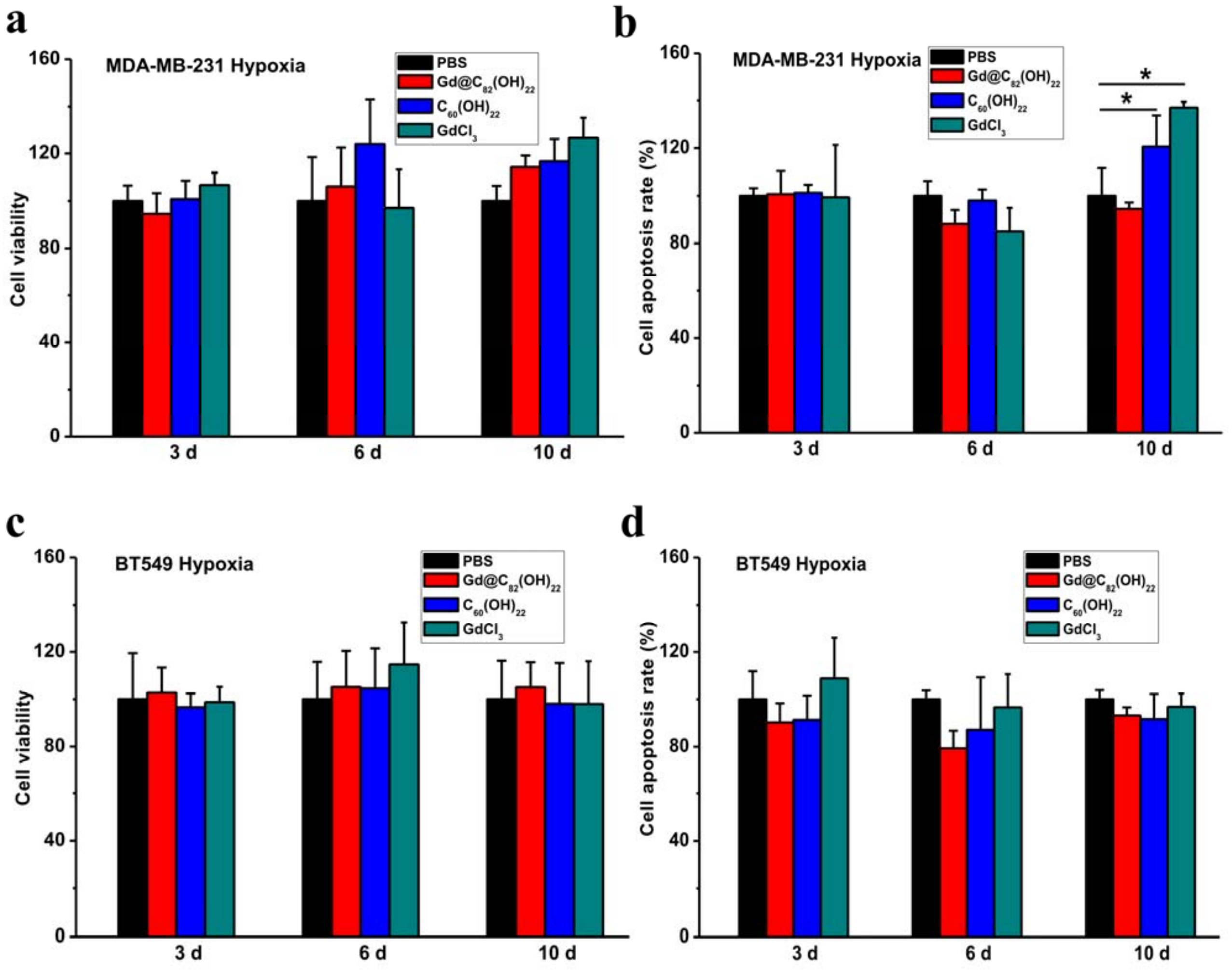

Supplementary Figure 12. MDA-MB-231 and BT549 cells were treated with PBS, Gd@$C_{82}(OH)_{22}$, $C_{60}(OH)_{22}$ or $GdCl_3$ (all 50 μM) for 10 days under hypoxia. (a, c) Cell viability was detected using CCK-8 assay. (b, d) Cells apoptosis analysis was detected using double staining with annexin V and PI. All the data are represented as mean ± s.e.m. (n = 3 each) with *$p < 0.05$ (one-way ANOVA, Tukey's post-hoc test).

a

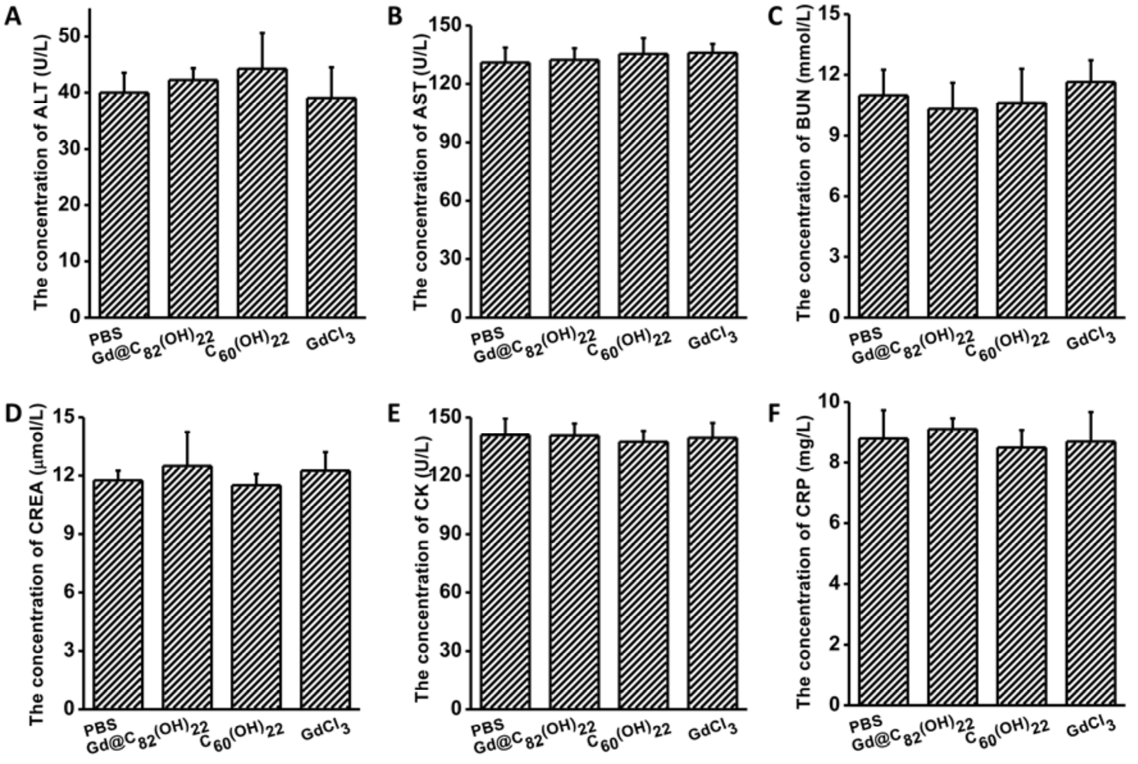

b

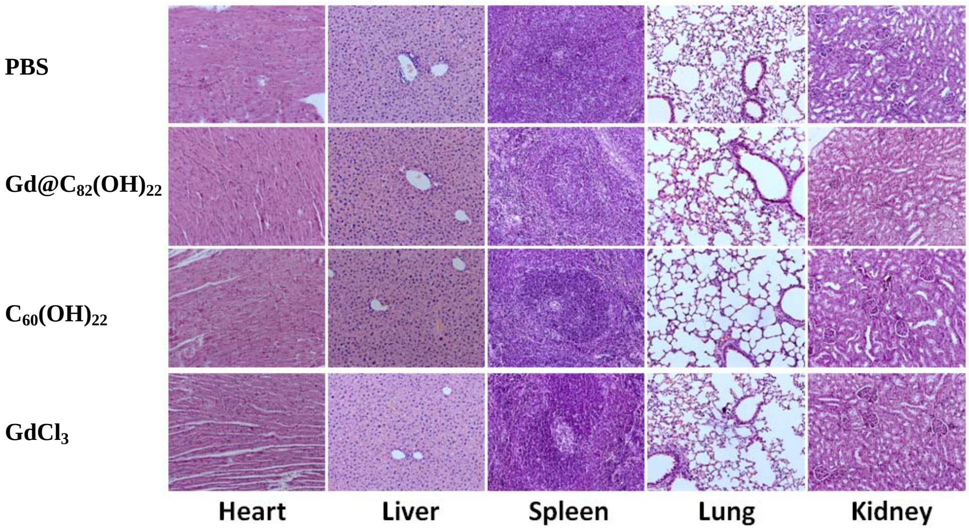

Supplementary Figure 13. Toxicity of $Gd@C_{82}(OH)_{22}$ nanoparticles *in vivo*. Nude mice were injected with either 0.1 mL PBS, or 2.5 μmol/kg $Gd@C_{82}(OH)_{22}$, $C_{60}(OH)_{22}$ or $GdCl_3$ for 21 days once a day *i.p.* before sacrifice. (a) Serum ALT (A), AST (B), BUN (C), CREA (D), CK (E) and CRP (F) levels. (b) HE staining of various tissues (liver, spleen, kidney, heart and lung).

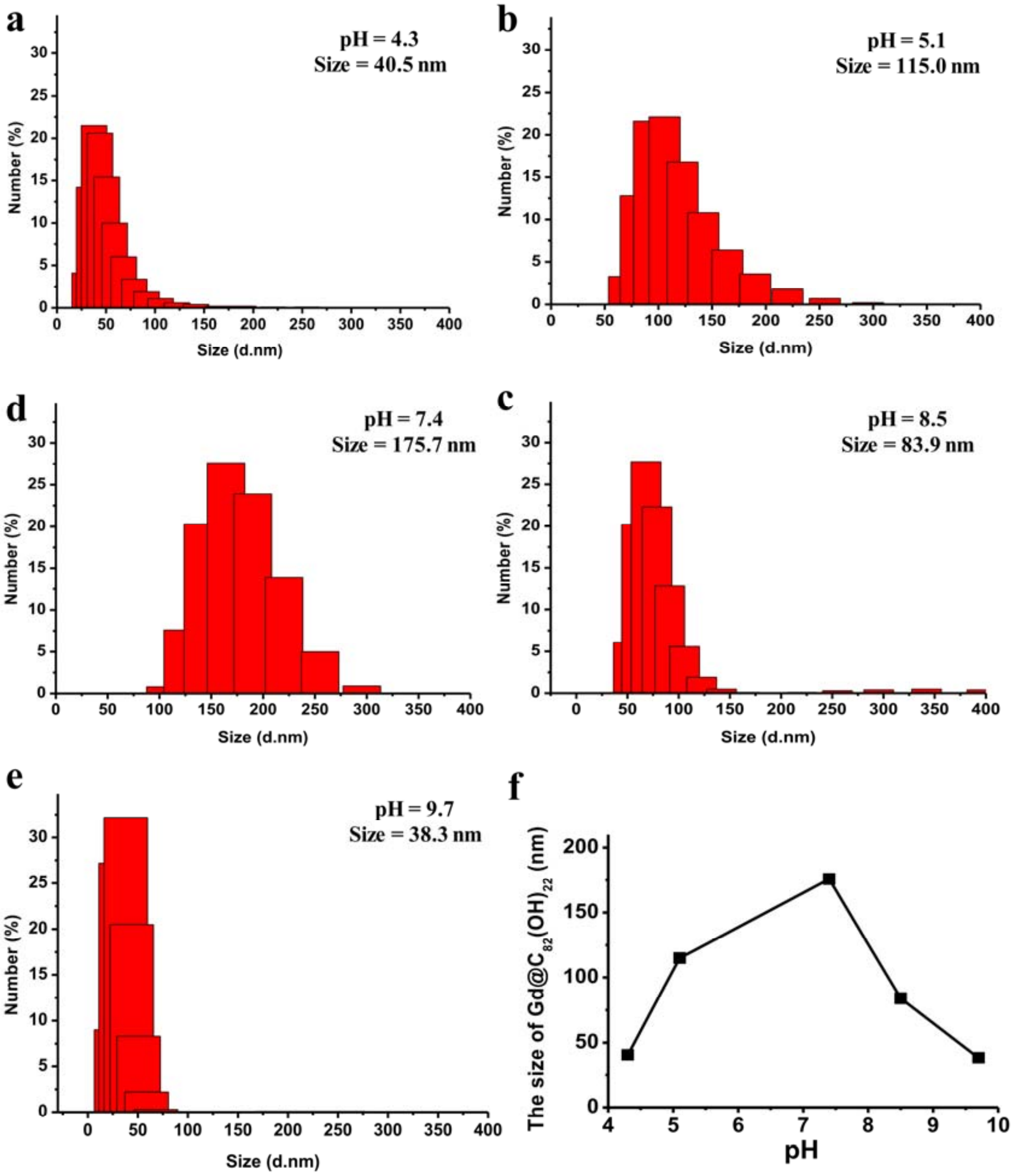

Supplementary Figure 14. The size and *zeta* potential measurement of the Gd@$C_{82}(OH)_{22}$ nanoparticle in different pH solutions. (a) pH = 4.3, the average size is ~40 nm; (b) pH = 5.1, the average size is ~116 nm; (c) pH = 7.4, the average size is ~175 nm; (d) pH = 8.5, the average size is ~84 nm; (e) pH = 9.7, the average size is 38 nm; (f) The average size of the Gd@$C_{82}(OH)_{22}$ nanoparticles in different pH solutions.

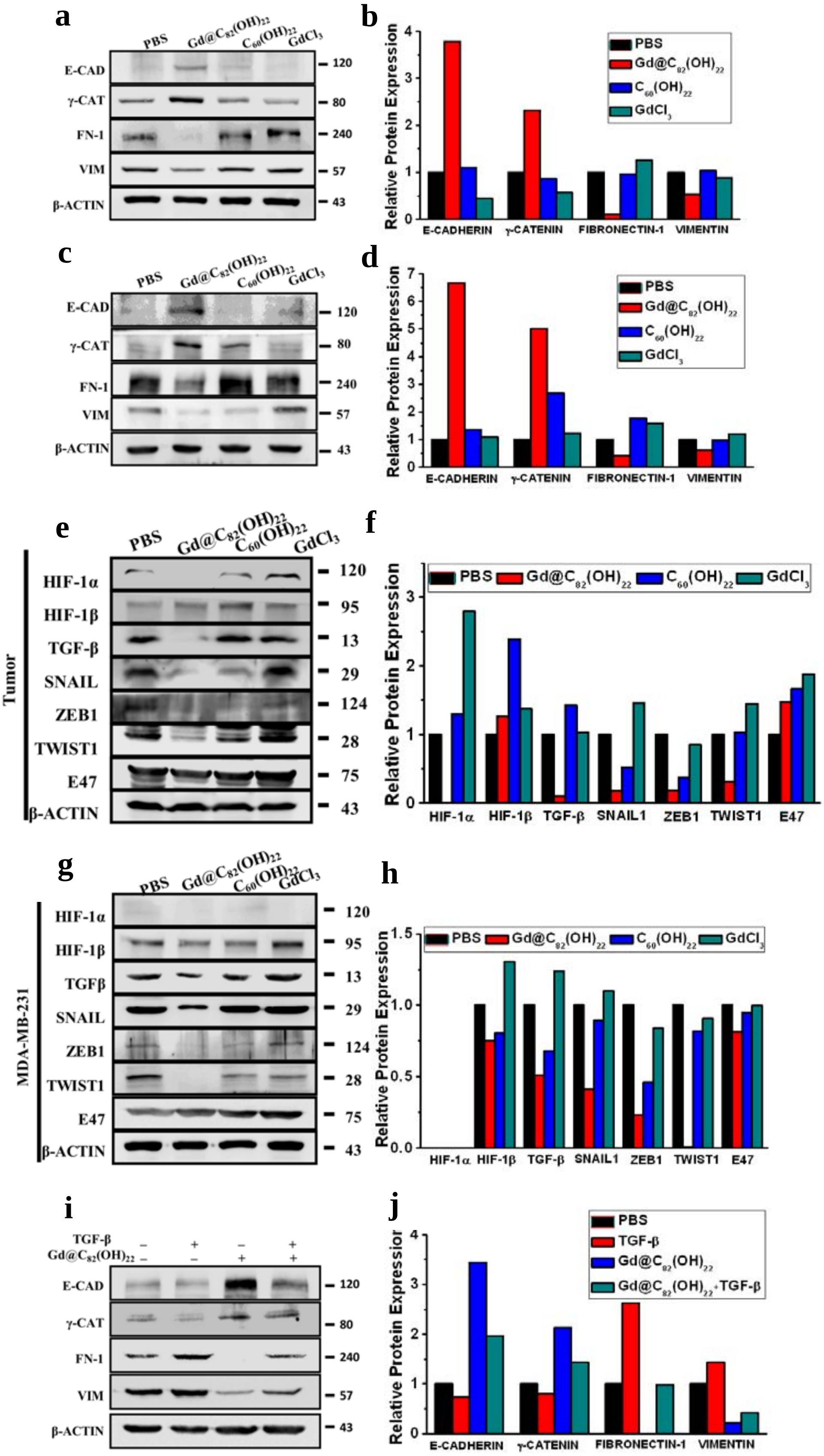
a
PBS
Gd@C82(OH)22
C60(OH)22
GdCl3
E-CAD
γ-CAT
FN-1
VIM
β-ACTIN
120
80
240
57
43
b
Relative Protein Expression
PBS
Gd@C82(OH)22
C60(OH)22
GdCl3
E-CADHERIN
γ-CATENIN
FIBRONECTIN-1
VIMENTIN
c
d
e
Tumor
HIF-1α
HIF-1β
TGF-β
SNAIL
ZEB1
TWIST1
E47
120
95
13
29
124
28
75
f
SNAIL1
g
MDA-MB-231
TGFβ
h
i
TGF-β
j
Gd@C82(OH)22+TGF-β

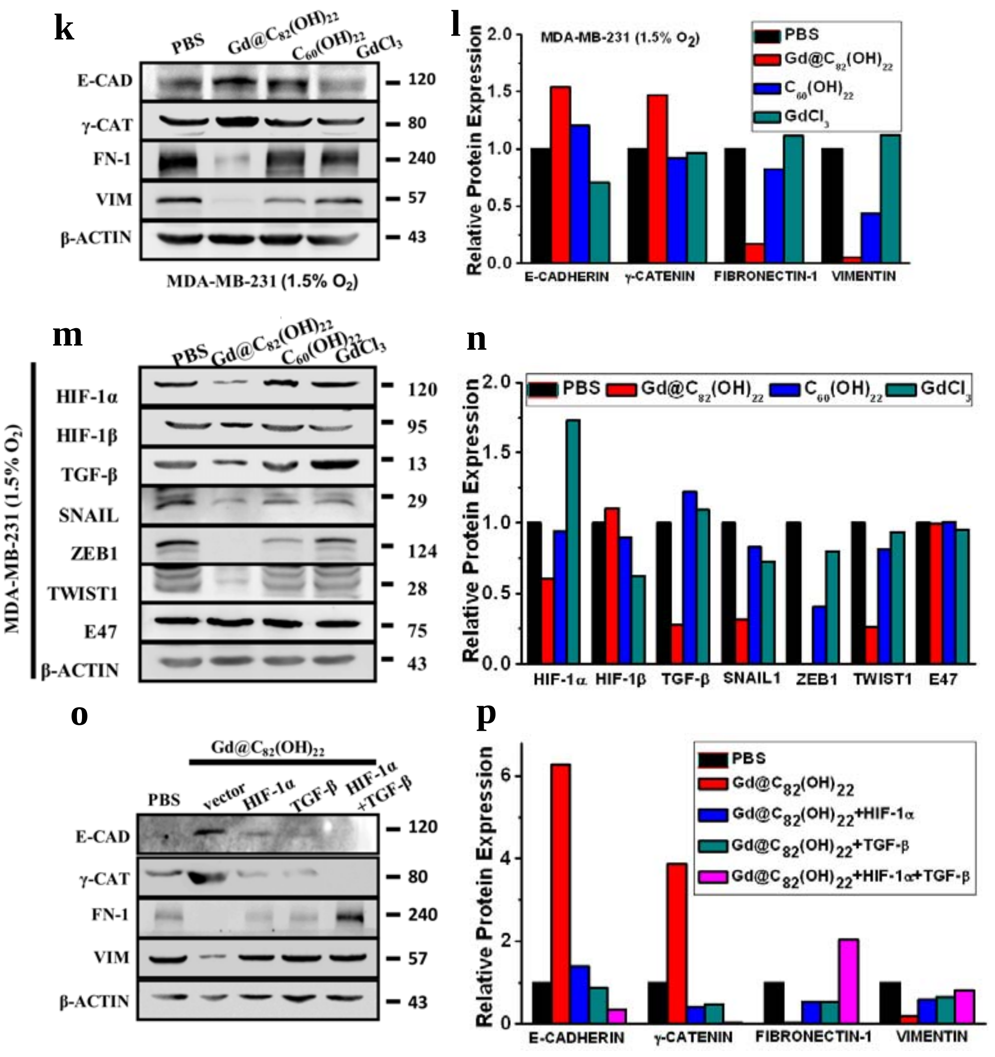

Supplementary Figure 15. All Western blot results have been quantified using Gelpro32 analysis software. (a, b) Figure 1d, (c, d) Figure 2g, (e, f) Figure 3c, (g, h) Figure 3j, (i, j) Figure 4c, (k, l) Figure 7e, (m, n) Figure 7i, (o, p) Figure 8b.

Figure 1d

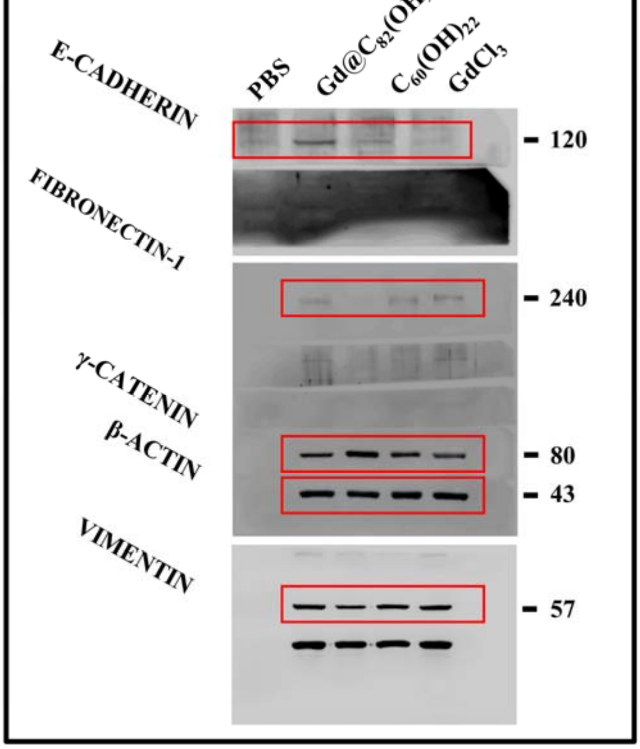

Figure 1k

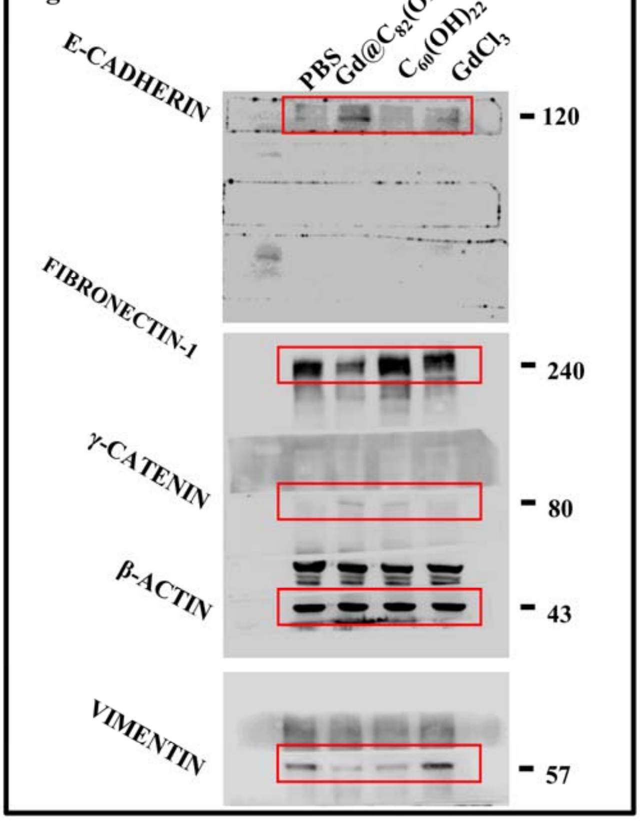

Figure 2d

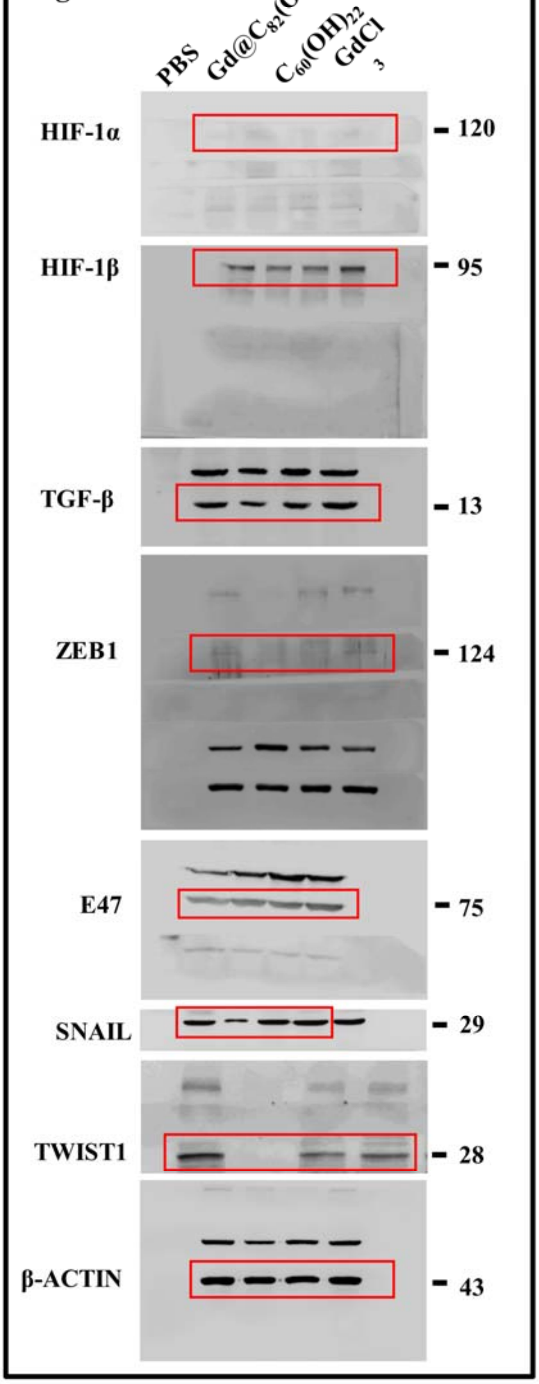

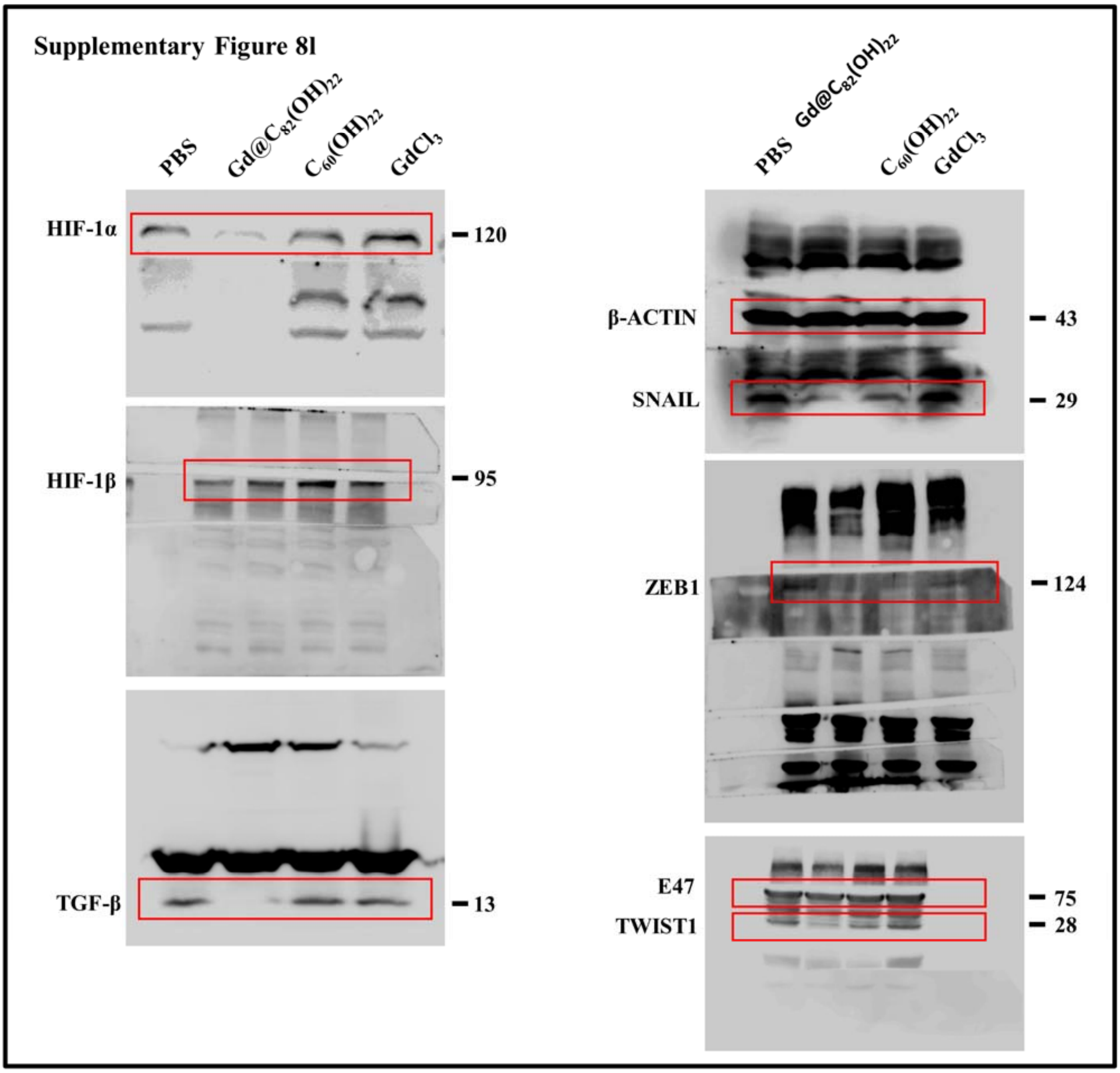

Supplementary Figure 8l
PBS
Gd@C82(OH)22
C60(OH)22
GdCl3
HIF-1α
120
HIF-1β
95
TGF-β
13
β-ACTIN
43
SNAIL
29
ZEB1
124
E47
75
TWIST1
28

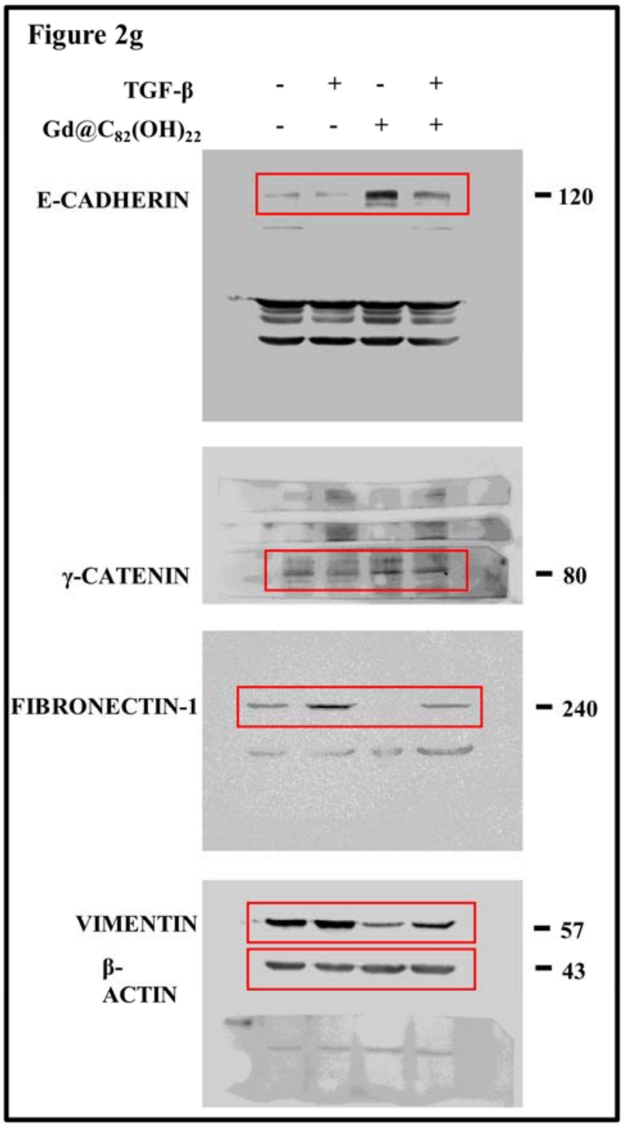
Figure 2g
TGF-β - + - +
Gd@C82(OH)22 - - + +
E-CADHERIN
120
γ-CATENIN
80
FIBRONECTIN-1
240
VIMENTIN
57
β-ACTIN
43

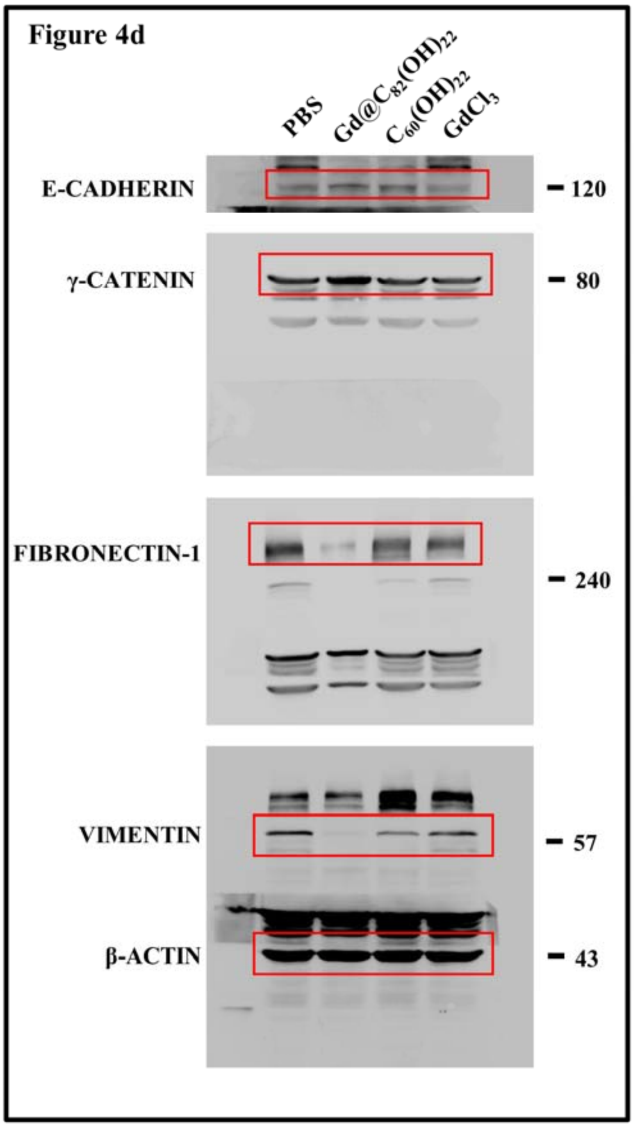
Figure 4d
PBS
Gd@C82(OH)22
C60(OH)22
GdCl3
E-CADHERIN
120
γ-CATENIN
80
FIBRONECTIN-1
240
VIMENTIN
57
β-ACTIN
43

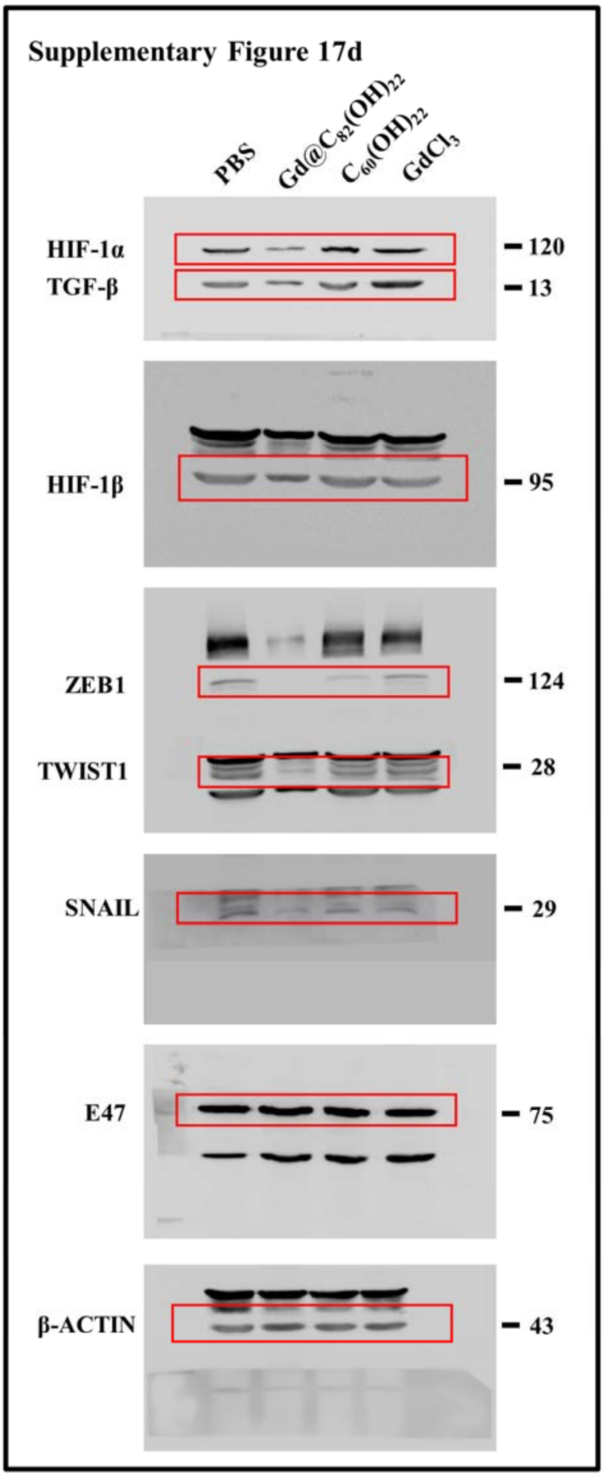

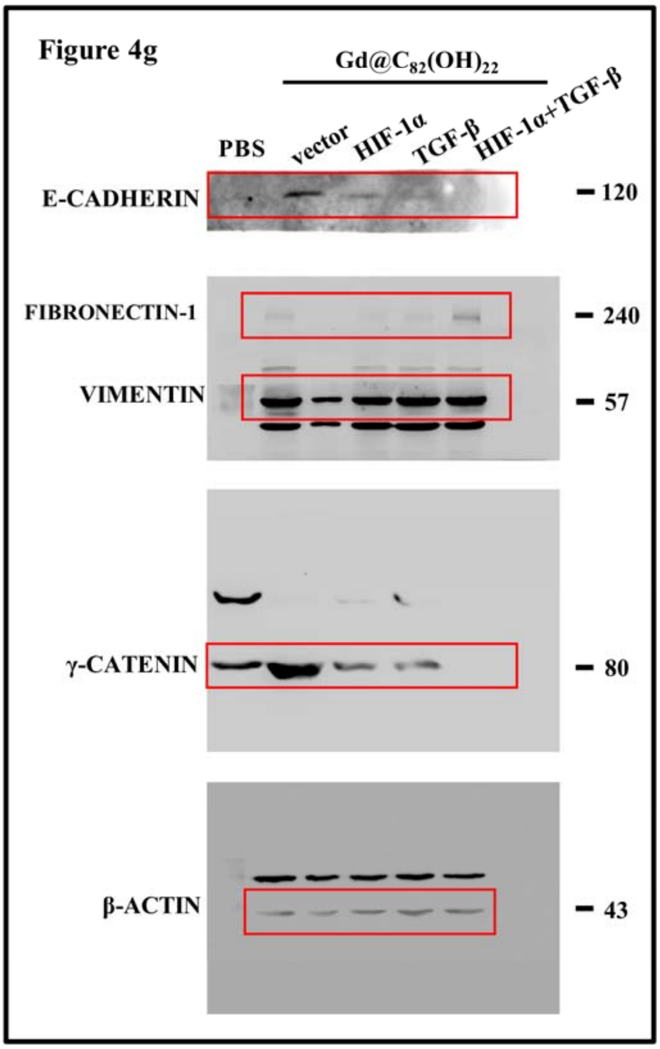

Supplementary Figure 16. Uncropped western blots with indicated areas of selection.

Supplementary Table 1. Anti-tumor activity of Gd@$C_{82}(OH)_{22}$ nanoparticles in mice model by approach I (early treatment).

Group	Tumor Weight (g)	T-test	Inhibition rate (%)
**PBS**	0.32 ± 0.25	/	/
**Gd@$C_{82}(OH)_{22}$**	0.18 ± 0.03	$p < 0.05$	55.25
**$C_{60}(OH)_{22}$**	0.19 ± 0.04	$p < 0.05$	40.62
**$GdCl_3$**	0.29 ± 0.20	$p > 0.05$	9.38

***** 1×10^6 MDA-MB-231 cells were injected *s.c.* and mice were treated with PBS, Gd@$C_{82}(OH)_{22}$, $C_{60}(OH)_{22}$ or $GdCl_3$ daily at day 0 for 21 days. And at the day 21st, the mice were executed and analysed.

Supplementary Table 2. Anti-tumor activity of Gd@$C_{82}(OH)_{22}$ nanoparticles in mice model by approach II (terminal treatment).

Group	Tumor Weight (g)	T-test	Inhibition rate (%)
**PBS**	0.45 ± 0.12	/	/
**Gd@$C_{82}(OH)_{22}$**	0.22 ± 0.10	$p < 0.05$	51.11
**$C_{60}(OH)_{22}$**	0.48 ± 0.12	$p > 0.05$	- 6.67
**$GdCl_3$**	0.45 ± 0.17	$p > 0.05$	0

***** 1×10^6 MDA-MB-231 cells were injected *s.c.* When the tumors grew to 100 mm^3, the mice were treated with PBS, Gd@$C_{82}(OH)_{22}$, $C_{60}(OH)_{22}$ or $GdCl_3$ daily for 21 days. And after 21 days, the mice were executed and analysed.

Supplementary Table 3. Incidence of lung and liver metastases in mice with MDA-MB-231 cells injection *i.v.* (metastasis model)

	**Lung metastases**	**Liver metastases**
**PBS***	6/6	4/6
**$Gd@C_{82}(OH)_{22}$***	1/6	0/6
**$C_{60}(OH)_{22}$***	5/6	3/6
**$GdCl_3$***	6/6	6/6

* 1×10^6 MDA-MB-231 cells were injected *i.v* and mice were treated with PBS, $Gd@C_{82}(OH)_{22}$, $C_{60}(OH)_{22}$ or $GdCl_3$ daily at day 0 for 21 days. And at the day 21st, the mice were executed and analysed.

* The numbers represent "the number of mice with lung or liver metastases/the number of mice in every group".

Supplementary Table 4. Summary of tumor formation in mice models

Groups		A	B	C	D	E	F	G	H	I
Injected cell number		5×10^6	5×10^5	5×10^4	5×10^3	5×10^2	1×10^6	1×10^6	1×10^6	1×10^6
Mice model		Cancer stem cells					Approach I	Approach II	Withdrawal	Metastasis
Delivery approach		Peritoneal	Peritoneal	Peritoneal	Peritoneal	Peritoneal	Peritoneal	Peritoneal	Peritoneal	Tail vein
	PBS	6	6	6	6	6	5	5	5	5
	$Gd@C_{82}(OH)_{22}$	6	6	6	6	6	5	5	5	5
	$C_{60}(OH)_{22}$	6	6	6	6	6	5	5	5	5
Injection	$GdCl_3$	6	6	6	6	6	5	5	5	5

Supplementary Table 5. Sequence of the oligonucleotides for real-time PCR experiments

Gene	Sense Strand (5'-3')	Antisense Strand (5'-3')	Amplicon (bp)
bmi1	AAATGCTGGAGAACTGGAAAG	CTGTGGATGAGGAGACTGC	124
csf1	GCAGCGGCTGATTGACAGT	CAGCTGCACAATGGCGAT	185
klf4	ACATGGCTGTCAGCGACGCG	GCCAGCGGTTATTCGGGGCAC	108
lin28a	TTCGGCTTCCTGTCCATGAC	TGACTCAAGGCCTTTGGAAG	123
nanog	CAACCAGACCCAGAACATCC	TTCCAAAGCAGCCTCCAAG	185
cd44	CAGGGACAGCTGCAGCCTCA	ACCTCGTCCCATGGGGTGTG	122
cd24	CCAACTAATGCCACCACCA	GACGTTTCTTGGCCTGAGTC	118
aldh1	TCGTCTGCTGCTGGCGACAATG	CCCAACCTGCACAGTAGCGCAA	113
ptgs2 (cox2)	TGAAACCCACTCCAAACACA	GAGAAGGCTTCCCAGCTTTT	185
tgf-β	GGAAATTGAGGGCTTTCGCC	CCGGTAGTGAACCCGTTGAT	90
hif1α	TTTTTCAAGCAGTAGGAATTGGA	GTGATGTAGTAGCTGCATGATCG	66
snail	TGCGCTACTGCTGCGCGAAT	GGGCTGCTGGAAGGTAAACTCTGGA	147
zeb1	AAGAATTCACAGTGGAGAGAAGCCA	CGTTTCTTGCAGTTTGGGCATT	50
e47	GCATTGAGGCCTTGTGGA	GGTAACGGTGGAGTCTCAGG	96
twist1	GGAGTCCGCAGTCTTACGAG	TCTGGAGGACCTGGTAGAGG	200
cdh1 (e-cadherin)	TGCCCAGAAAATGAAAAAGG	GTGTATGTGGCAATGCGTTC	199
jup (γ-catenin)	CCCCGCCTGGTGCAGAACTG	GGAGAGTGTGCCCGTGGCAC	153
vim (vimentin)	CGAGGAGAGCAGGATTTCTC	GGTATCAACCAGAGGGAGTGA	90
fn-1(fibronectin-1)	AGGCAGGCTCAGCAAATG	TTAGGACGCTCATAAGTGTCACCC	260

cdh2 (n-cadherin)	ACAGTGGCCACCTACAAAGG	CCGAGATGGGGTTGATAATG	200
ocln (occludin)	TGCCGCGTTGGTGATCTTT	GCCCAGGATAGCACTCACTATT	100
ctnna1 (α-catenin)	CCATGCAGGCAACATAAACTTC	GGCTCCAACAGTCTCTCAACT	81
ctnnb1 (β-catenin)	CCCACTGGCCTCTGATAAAGG	ACGCAAAGGTGCATGATTTG	80
il-6	GGATTCAATGAGGAGACTTGCC	TGGCATTTGTGGTTGGGTCA	208
il-8	TAAAGACATACTCCAAACC	ACTTCTCCACAACCCTC	165
vegf	CGCAAGAAATCCCGGTATAA	TCTCCGCTCTGAGCAAGG	111
mmp-2	ACAGAACCCTTGGAGCCAAT	GAAAGGTTCTAAGGCAGCCA	142
mmp-9	CGTCTTCCAGTACCGAGAGA	GCAGGATGTCATAGGTCACG	119
gapdh	TCCCATCACCATCTTCCAGG	CCATCACGCCACAGTTTCC	98

Supplementary Table 6. List of proteins tested by antibodies and characteristics of the corresponding antibodies used

Protein	Assay	Antibody	Origin	Dilution	Incubation period
E-CADHERIN	WB	mmab	# 610181, BD	1:5000	overnight
E-CADHERIN	IHC	mmab	# 610181, BD	1:50	overnight
E-CADHERIN	IF	mmab	# 610181, BD	1:100	overnight
γ-CATENIN	WB	mmab	#sc-8415, Santa Cruz	1:1000	overnight
γ- CATENIN	IHC	mmab	#sc-8415, Santa Cruz	1:50	overnight
γ- CATENIN	IF	mmab	#sc-8415, Santa Cruz	1:50	overnight
FIBRONECTIN-1	WB	mmab	# 610077, BD	1:5000	overnight
FIBRONECTIN-1	IHC	mmab	# 610077, BD	1:200	overnight
FIBRONECTIN-1	IF	mmab	# 610077, BD	1:200	overnight
VIMENTIN	WB	mmab	# 550513, BD	1:5000	overnight
VIMENTIN	IHC	mmab	# 550513, BD	1:100	overnight
VIMENTIN	IF	mmab	# 550513, BD	1:200	overnight
TWIST1	WB	rpab	#sc-15393, Santa Cruz	1:1000	overnight
TWIST1	IHC	rpab	#sc-15393, Santa Cruz	1:50	overnight
SNAIL	WB	rpab	#sc-28199, Santa Cruz	1:1000	overnight
SNAIL	IHC	rpab	#sc-28199, Santa Cruz	1:100	overnight
ZEB1	WB	rpab	#sc-25388, Santa Cruz	1:1000	overnight
ZEB1	IHC	rpab	#sc-25388, Santa Cruz	1:50	overnight
E47	WB	mmab	# 554199, BD	1:5000	overnight

E47	IHC	mmab	# 554199, BD	1:50	overnight
COX2	WB	gpab	#sc-23984, Santa Cruz	1:1000	overnight
COX2	IHC	gpab	#sc-23984, Santa Cruz	1:50	overnight
TGF-β	WB	rpab	#sc-146, Santa Cruz	1:1000	overnight
TGF-β	IHC	rpab	#sc-146, Santa Cruz	1:50	overnight
HIF1α	WB	mmab	# 610958, BD	1:5000	overnight
HIF1α	IHC	mmab	# 610958, BD	1:50	overnight
HIF1β	WB	mmab	# 611078, BD	1:5000	overnight
HIF1β	IHC	mmab	# 611078, BD	1:200	overnight
β-ACTIN	WB	mmab	#M20010, Abmart	1:5000	1.5 hour

Abbreviations: WB, Western blot; IHC, immunohistochemistry; IF, immunofluorescence; mmab, mouse monoclonal antibody; rpab, rabbit polyclonal antibody; gpab, goat polyclonal antibody.
